# Antimicrobial potential of *Miscanthus* × *giganteus* extracts: linking *in vitro* and *in silico* activity with *in situ* efficacy in milk

**DOI:** 10.1039/d6ra07636j

**Published:** 2026-09-25

**Authors:** Dragana Robajac, Dejan Stojković, Uroš Gašić, Gokhan Zengin, Mehmet Veysi Cetiz, Jovana Petrović

**Affiliations:** a Institute for the Application of Nuclear Energy (INEP), University of Belgrade Belgrade Serbia draganar@inep.co.rs; b Institute for Biological Research “Siniša Stanković” (IBISS), National Institute of the Republic of Serbia, University of Belgrade Belgrade Serbia dejanbio@ibiss.bg.ac.rs uros.gasic@ibiss.bg.ac.rs jovana0303@ibiss.bg.ac.rs; c Department of Biology, Science Faculty, Selcuk University, Campus-Konya Turkey gokhanzengin@selcuk.edu.tr; d Department of Medical Biochemistry, Faculty of Medicine, Harran University Sanliurfa Turkey mvcetiz@gmail.com

## Abstract

*Miscanthus* × *giganteus* is a high-biomass, low-input crop whose potential as a source of antimicrobial compounds remains insufficiently explored. This study investigated the phytochemical composition, antioxidant and antimicrobial properties of methanolic (MeOH) and dichloromethane (DCM) extracts obtained from roots, rhizomes, stems, and leaves, integrating *in vitro* assays with molecular modelling and validation in a food matrix. Targeted UHPLC-MS/MS analysis revealed marked organ- and solvent-dependent differences in phenolic composition, with *p*-coumaric acid predominating in roots and rhizomes, while leaves were particularly enriched in 5-*O*-caffeoylquinic acid and isoorientin. MeOH root extract exhibited the strongest overall antioxidant potential, consistent with its high phenolic content. The extracts displayed broad antimicrobial activity against foodborne and pathogenic bacteria, filamentous fungi, and *Candida* spp., with antibacterial MIC and MBC values of 0.23–1.88 and 0.47–3.75 mg mL^−1^, respectively, and activities frequently comparable or superior to commercial food preservatives. Molecular docking, 100 ns molecular dynamics simulations, and MM-PBSA analyses identified isoorientin as a particularly promising antimicrobial constituent, showing stable interactions with microbial targets, including *Bacillus cereus* DltA. Guided by the phytochemical, antimicrobial, and *in silico* findings, the MeOH leaf extract was further evaluated against *B. cereus* in milk. At 2 mg mL^−1^, bacterial growth was inhibited by 97% after 24 h and remained suppressed by 81% after 48 h. These findings identify *M.* × *giganteus* as a sustainable source of bioactive compounds and demonstrate that its antimicrobial activity can be retained in a complex food matrix, supporting further development of *Miscanthus*-derived natural preservation strategies.

## Abstract 2

*M.* × *giganteus* as a sustainable source of bioactive compounds in the development of natural preservation strategies.

## Graphical abstract

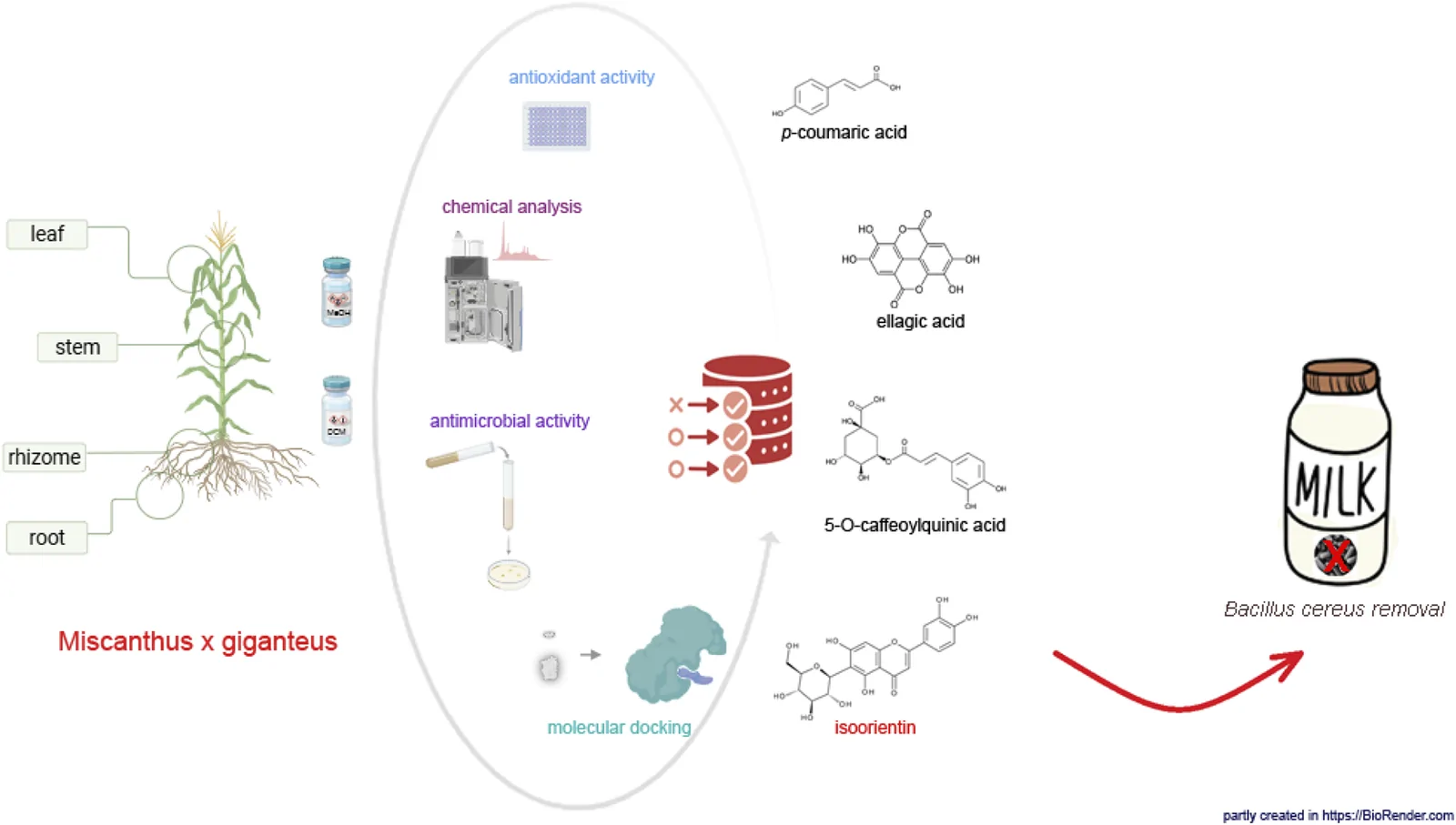

## 1 Introduction

*Miscanthus* spp. (fam. Poaceae) are widely distributed in Africa, Eurasia, and the Pacific on various soil types with minimum maintenance required, even under drought conditions. This valuable source of lignocellulosic biomass can be used in the production of paper, building supplies, geotextiles *etc.* Thanks to the high biomass yield and substantial lignin content, low-input crops (including *Miscanthus* spp.) may be successfully exploited in the production of biocomposites and packaging materials.^1^ In addition to their industrial applications, recent studies have indicated that *Miscanthus* spp. may also serve as a source of bioactive secondary metabolites, including phenolic compounds with antioxidant and antimicrobial properties.^2^ In this context, the control of foodborne pathogens in real food systems is essential for the effective valorization of plant-derived bioactive compounds. Among these microorganisms, *Bacillus cereus* represents a major concern in dairy industry due to its ability to form heat resistant spores and persist throughout different stages of production and processing. Its resilience in food matrices limits the effectiveness of usually applied preservation techniques, highlighting the need for alternative antimicrobial agents. Plant derived phenolic compounds have attracted attention due to their ability to inhibit microbial growth through multiple mechanisms, including membrane disruption and enzyme inhibition.^3^

Considering the emerging potential of *M.* × *giganteus* as a source of bioactive compounds, the present study aimed to characterize the phytochemical composition of methanolic (MeOH) and dichloromethane (DCM) extracts obtained from different plant organs and to evaluate their antioxidant and antimicrobial activities. The use of solvents with different polarities enabled the extraction of chemically diverse metabolites, ranging from hydrophilic phenolic acids and flavonoid glycosides to more lipophilic phenolic derivatives. Furthermore, molecular docking and molecular dynamics simulations were performed to investigate potential mechanisms underlying antibacterial activity. To ensure relevance to food applications, the most biologically active extract was further evaluated in *B. cereus* inoculated milk, providing insight into its effectiveness as a natural preservative in a real food system.

## 2 Materials and methods

### 2.1. Plant material extraction

*M*. × *giganteus* was harvested in August 2017 from the experimental field of INEP, University of Belgrade, Serbia (voucher specimen Mg_INEP_01). Roots, rhizomes, stems, and leaves were separated, air-dried, and extracted with methanol (MeOH) or dichloromethane (DCM) following Vaz *et al.*^4^ Extracts were obtained by overnight extraction, sonication, centrifugation, and filtration, and the procedure was repeated three times. Combined extracts were evaporated to dryness and stored at 4 °C until further analyses.

### 2.2. UHPLC(−)HESI-QqQ-MS/MS targeted metabolomics analysis

Content of some specific phenolics was analyzed using Dionex Ultimate 3000 UHPLC system (Thermo Fisher Scientific, Bremen, Germany) connected to a triple-quadrupole (QqQ) MS (TSQ Quantum Access Max, Thermo Fisher Scientific, Basel, Switzerland). The analytical column Syncronis C18 aqua (100 × 2.1 mm) with 1.7 µm particle size (Thermo Fisher Scientific, USA) was used for chromatographic separation. The mobile phase consisted of (A) water + 0.1% formic acid (v/v), and (B) 100% acetonitrile (MS grade) + 0.1% formic acid (v/v), which were applied in the following gradient elution: 0–2.0 min 5% B, 2.0–14.0 min 5–95% B, 14.0–14.2 min from 95% to 5% B, and 5% B until the 20th min. The flow rate was set to 0.3 mL min^−1^. The mass detector was equipped with heated electrospray ionization (HESI) source operated in the negative ionization mode. Samples were dissolved in methanol (20 mg mL^−1^). The parameters regarding flow rate, mobile phase composition, HESI source, mass detector settings, as well as gradient elution program were as in Ivanov *et al.*^5^ Multiple mass spectrometric scanning modes, including full scanning, product ion scanning (PIS) and neutral loss scanning were conducted for the qualitative analysis. A selected reaction monitoring experiment for quantitative analysis was performed using two MS^2^ fragments for each compound, which were previously defined as dominant in PIS experiments. The phenolics were identified by comparison with commercial standards (Sigma-Aldrich, Steinheim, Germany). Total amounts of each compound were evaluated by calculating the peak areas. Retention times and MS data (molecular ion and major MS^2^ fragments with collision energies) of tested compounds are given in Supplementary file (Table S1).

### 2.3. Antioxidant assays

Total antioxidant capacity (TAC) was measured according to Prieto *et al.* (1999) and expressed as mg of Trolox equivalents per gram of dry extract (mg TE g^−1^). The reducing power of extracts was examined using cupric reducing antioxidant power (CUPRAC) assay as described in Nurcholis *et al.*^6^ and expressed as mg of ascorbic acid equivalents (mg AAE/g). Potassium ferricyanide reducing power (FRAP) of extracts was analyzed as in Mokrani *et al.*^7^ and results expressed as mg of ascorbic acid equivalent (mg AAE g^−1^). DPPH scavenging assay was performed following the procedure described by Vaz *et al.*^4^ Total phenolic content (TPC) was determined according to Mokrani *et al.*^7^ and expressed as mg of gallic acid equivalents (mg GAE g^−1^). Total flavonoid content (TFC) was determined according to Nurcholis *et al.*^6^ and expressed as mg of quercetin equivalents (mg QE g^−1^).

### 2.4. Antimicrobial activity

Antimicrobial activity was evaluated against Gram-positive bacteria (*Staphylococcus aureus* ATCC 11632, *Bacillus cereus* food isolate, *Listeria monocytogenes* NCTC 7973), Gram-negative bacteria (*Escherichia coli* ATCC 25922, *Enterobacter cloacae* ATCC 35030, *Salmonella enterica* serovar Typhi ATCC 13311), filamentous fungi (*Aspergillus fumigatus* ATCC 9197, *A. niger* ATCC 6275, *A. versicolor* ATCC 11730, *Penicillium funiculosum* ATCC 36839, *P. verrucosum* var. *cyclopium* food isolate, and *Trichoderma viride* IAM 5061), and yeasts (*Candida albicans* 475/15, 13/15, and 17/15, *C. parapsilosis* ATCC 22019, *C. tropicalis* ATCC 750, and *C. krusei* H1/16). All strains are deposited in the Mycological Laboratory at IBISS. Antimicrobial activity was determined by the broth microdilution method as described by Ivanov *et al.*^5^ MIC was defined as the lowest concentration inhibiting visible growth, while MBC/MFC corresponded to the lowest concentration causing 99.5% reduction of the initial inoculum. Fungal suspensions (1 × 10^5^ cells per mL) were prepared in 0.85% saline containing 0.1% Tween 80 and incubated with serially diluted samples in Malt broth at 25 °C for 5 days. E211 and E224 (10 mg mL^−1^) were used as antibacterial and antifungal positive controls, whereas ketoconazole (1 mg mL^−1^) served as the positive control for *Candida* spp.

#### 2.4.1. Antimicrobial activity of *M. Giganteus* leaf extract against *B. cereus* in milk

Antimicrobial activity of leaf methanol extract against *B. cereus* was evaluated in commercial sample of milk using procedure described by Wei *et al.*^8^ with some modifications. Sterile milk samples were inoculated with bacterial suspension (10^5^ CFU mL^−1^) and treated with two concentrations of the extract (1 mg mL^−1^ and 2 mg mL^−1^), while untreated and solvent-treated samples served as controls. Samples were incubated at 37 °C and bacterial counts were determined after 24 h and 48 h. Serial decimal dilutions were prepared, and aliquots (0.1 mL) were spread on agar plates in triplicate. After incubation, colonies were counted and growth inhibition percentages were calculated relative to the untreated control, following the formula:
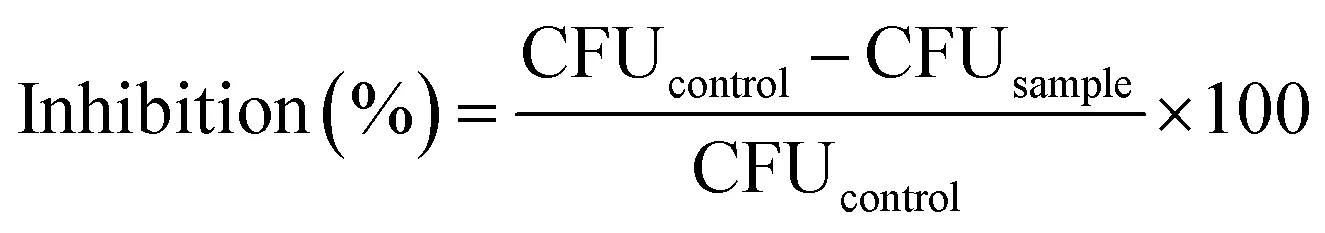


### 2.5. Molecular modelling

Details regarding protein and ligand preparation, docking grid and parameters, validation and interaction analysis, molecular dynamics simulations and MM/PBSA free energy calculation are given in Supplementary file.

### 2.6. Statistical analysis

Results of targeted metabolomics analysis and antioxidant assays are expressed as mean ± standard deviation of three measurements. Data distribution was tested using Shapiro–Wilk test. The correlation between the total phenolic and flavonoid content of prepared extracts with obtained antioxidant activity was assessed using Pearson's r; *p* value < 0.05 was taken as significant. The data was analyzed using GraphPad Prism 9.0.0. (GraphPad Software, Inc., San Diego, CA).

## 3 Results

### 3.1. Chemical analysis

Overall, the phenolic composition showed considerable differences among plant organs (root, rhizome, stem and leaf) as well as between used solvents (Table 1). MeOH extracts generally contained substantially higher amounts of the identified phenolic compounds than the corresponding DCM extracts. Based on the sum of the quantified compounds, the MeOH root extract showed the highest overall phenolic content (22 763.86 µg g^−1^), followed by the rhizome (9312.82 µg g^−1^), stem (5925.43 µg g^−1^), and leaf (4873.16 µg g^−1^) extracts. Contrary to this, the total amounts detected in DCM extracts were considerably lower, ranging from 235.76 µg g^−1^ in leaves to 604.30 µg g^−1^ in rhizomes. Overall, DCM extraction yielded considerably lower amounts of most of the quantified phenolics.

**Table 1:** UHPLC(−)HESI-QqQ-MS/MS targeted analysis of phenolic compounds in *M.* × *giganteus* MeOH and DCM extracts. Data are expressed as mean (µg of tested compound per g of dry extract) with standard deviation

	Root		Rhizome		Stem		Leaf	
Concentration (µg g^−1^)	MeOH	DCM	MeOH	DCM	MeOH	DCM	MeOH	DCM
3-*O*-Caffeoylquinic acid	12.68 ± 0.45	0	387.86 ± 13.63	0.38 ± 0.01	7.97 ± 0.28	4.00 ± 0.14	749.93 ± 26.35	0.28 ± 0.01
5-*O*-Caffeoylquinic acid	82.60 ± 4.42	1.04 ± 0.06	2513.64 ± 134.64	1.20 ± 0.06	48.31 ± 2.59	26.98 ± 1.45	2864.49 ± 153.43	2.71 ± 0.15
Caffeic acid	7.01 ± 0.15	1.26 ± 0.03	61.85 ± 1.29	2.23 ± 0.05	14.31 ± 0.30	1.28 ± 0.03	8.65 ± 0.18	1.63 ± 0.03
Ellagic acid	106.70 ± 9.37	59.47 ± 5.23	120.37 ± 10.58	63.23 ± 5.56	82.80 ± 7.27	52.08 ± 4.58	27.70 ± 2.43	189.53 ± 16.65
*p*-Coumaric acid	22 511.64 ± 607.01	313.44 ± 8.45	6217.87 ± 167.66	532.40 ± 14.36	5729.16 ± 154.48	493.48 ± 13.31	115.98 ± 3.13	16.67 ± 0.45
Isoorientin	20.65 ± 0.78	0	3.90 ± 0.15	0	6.79 ± 0.26	1.23 ± 0.05	937.07 ± 35.19	14.69 ± 0.55
Rutin	0	0.20 ± 0.01	0	0.38 ± 0.02	0.45 ± 0.02	0.30 ± 0.01	0	0.43 ± 0.02
Vitexin	2.53 ± 0.06	0	0.59 ± 0.01	0	3.27 ± 0.07	0	134.80 ± 3.01	1.68 ± 0.04
Quercetin-3-*O*-glucoside	1.75 ± 0.08	3.15 ± 0.15	2.51 ± 0.12	3.26 ± 0.15	1.97 ± 0.09	3.28 ± 0.15	6.82 ± 0.32	3.10 ± 0.14
Isorhamnetin-3-*O*-rutinoside	0	0	0	0	0	0	22.95 ± 0.48	0
Kaempferol-3-*O*-glucoside	1.83 ± 0.02	0.51 ± 0.01	2.13 ± 0.03	0.63 ± 0.01	2.21 ± 0.03	0	0	1.49 ± 0.02
Eriodictyol	4.73 ± 0.01	1.15 ± 0.01	0	0	3.14 ± 0.01	1.90 ± 0.01	2.42 ± 0.01	1.33 ± 0.01
Luteolin	7.58 ± 0.17	0	0	0	0	0	0	0
Naringenin	1.59 ± 0.07	0.91 ± 0.04	1.24 ± 0.06	0	1.70 ± 0.08	0	1.49 ± 0.07	0
Hispidulin	2.57 ± 0.05	6.92 ± 0.15	0.86 ± 0.02	0.59 ± 0.01	23.35 ± 0.49	0.88 ± 0.02	0.86 ± 0.02	2.22 ± 0.05

**Fig. 1:**
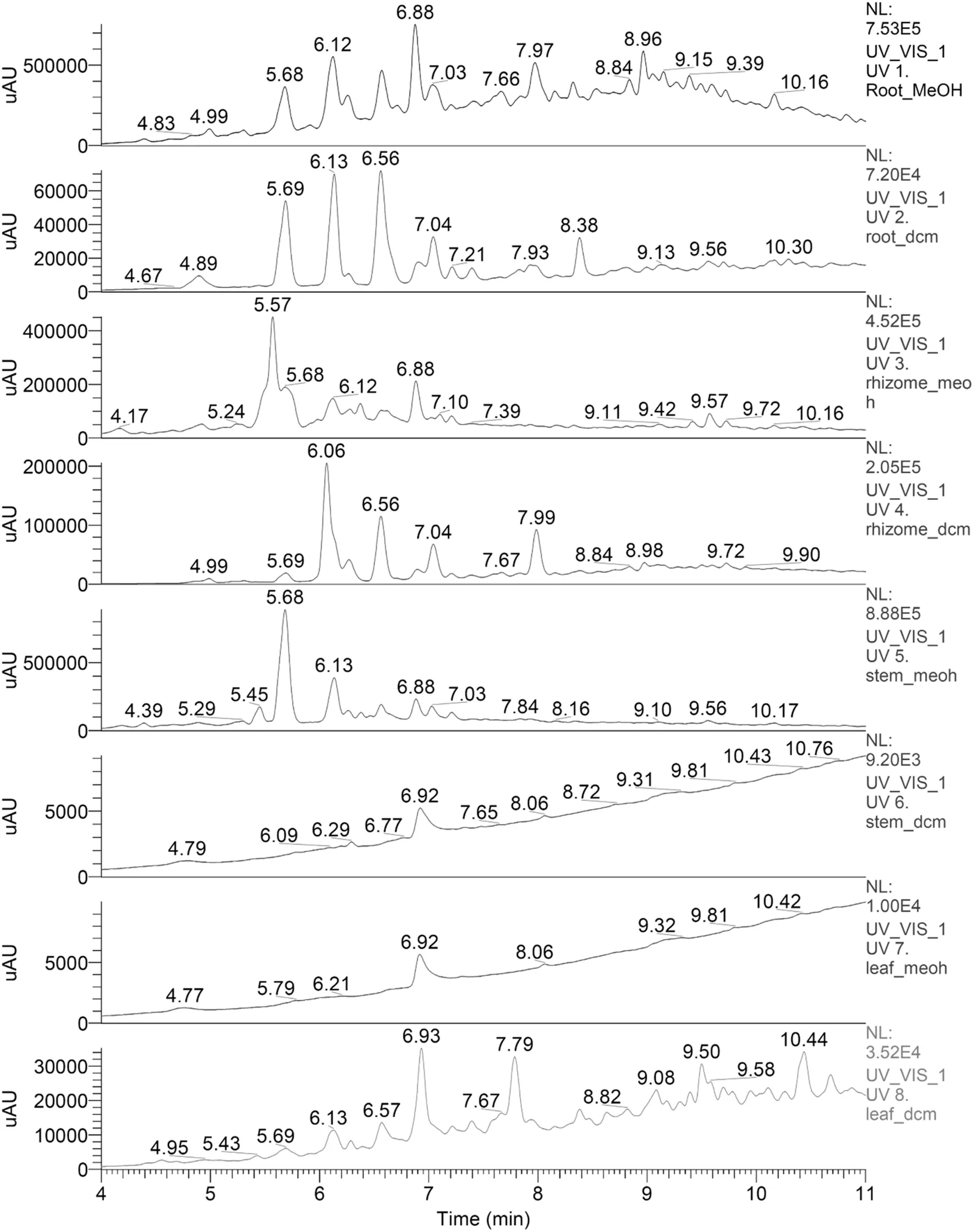
Representative chromatograms obtained after UHPLC(−)HESI-QqQ-MS/MS targeted metabolomics analysis of tested samples.

Analyzed phenolic compounds also differed considerably among plant organs (Fig. 1). Phenolic acids were dominant in MeOH extracts of root and stem, whereas rhizome extracts contained high amounts of both *p*-coumaric acid and caffeoylquinic acids (6217.87 and 2901.5 µg g^−1^). MeOH leaf extract showed a distinct profile, characterized by high levels of 3-*O*- and 5-*O*-caffeoylquinic acids (749.93 µg g^−1^ and 2864.49 µg g^−1^, respectively), together with flavonoids isoorientin (937.07 µg g^−1^) and vitexin (134.80 µg g^−1^). Some compounds were limited to a specific organ, *i.e.* isorhamnetin-3-*O*-rutinoside was detected exclusively in the MeOH leaf extract (22.95 µg g^−1^), whereas luteolin was detected only in the MeOH root extract (7.58 µg g^−1^).

As for the individual compounds, *p*-coumaric acid was the predominant constituent, particularly in the MeOH extracts. Its highest concentration was recorded in roots (22 511.64 µg g^−1^), followed by rhizomes (6217.87 µg g^−1^), stems (5729.16 µg g^−1^), and leaves (115.98 µg g^−1^). In the DCM extracts, *p*-coumaric acid was also abundant, although at considerably lower concentrations, with the highest level found in rhizomes (532.40 µg g^−1^), followed by stems (493.48 µg g^−1^), roots (313.44 µg g^−1^), and leaves (16.67 µg g^−1^). The highest amount of 5-*O*-caffeoylquinic was detected in leaves (2864.49 µg g^−1^) and rhizomes (2513.64 µg g^−1^); 3-*O*-caffeoylquinic acid showed a similar distribution, with 749.93 and 387.86 µg g^−1^ in leaves and rhizomes, respectively. Among the identified flavonoids, isoorientin (937.07 µg g^−1^) and vitexin (134.80 µg g^−1^) were especially abundant in the MeOH leaf extract. Rutin was detected only at trace levels (<1 µg g^−1^) in the DCM extracts, which is consistent with its poor extraction by a relatively non-polar solvent.

### 3.2. Antioxidant activity

Comprehensive analysis of *M.* × *giganteus* extracts revealed a wide range of antioxidant properties in all tested samples (Table 2). Extracts were rich in phenolic compounds, with the highest concentration detected in MeOH root extract (87.0 mg GAE g^−1^), whereas leaf DCM extract had the highest flavonoid content (20.6 mg QAE g^−1^). The highest TAC activity was observed in the MeOH root extract (594.9 mg TE g^−1^), followed by MeOH leaves and rhizomes extracts (395.3 and 377.7 mg TE g^−1^, respectively). In CUPRAC assay, DCM root extract was the most active (51.2 mg AAE g^−1^), followed by MeOH leaf (45.8 mg AAE g^−1^), DCM stem (40.8 mg AAE g^−1^), and MeOH rhizome (32.6 mg AAE g^−1^) extract. The highest reducing potential measured with FRAP assay was observed with root MeOH and leaf MeOH extracts (50.3 and 34.7 mg AAE g^−1^). The strongest DPPH activity was observed in MeOH root extract, followed by the MeOH leaves extract (EC_50_ 0.6 and 0.9 mg mL^−1^).

**Table 2:** Antioxidant activity of *M.* × *giganteus* extracts. Data are expressed as a means with standard deviation in milligram of equivalent per gram of dry extract

	FRAP(mg AAE g^−1^)	CUPRAC(mg AAE g^−1^)	TAC (mg TE g^−1^)	DPPH(EC_50_ mg mL^−1^)	TPC(mg GAE g^−1^)	TFC (mg QE g^−1^)
Root MeOH	50.3 ± 3.5	29.4 ± 0.5	594.9 ± 1.6	0.6 ± 0.10	87.0 ± 0.2	1.7 ± 0.2
Root DCM	9.5 ± 0.4	51.2 ± 0.5	115.4 ± 5.5	12.4 ± 1.50	13.2 ± 1.4	9.0 ± 0.5
Rhizome MeOH	25.5 ± 5.5	32.6 ± 2.5	377.7 ± 0.8	1.7 ± 0.10	32.8 ± 4.2	0.5 ± 0.1
Rhizome DCM	11.2 ± 0.1	29.7 ± 0.1	171.9 ± 0.6	6.4 ± 0.30	18.4 ± 3.4	5.4 ± 0.1
Stem MeOH	21.8 ± 5.9	22.1 ± 2.9	300.1 ± 22.3	2.5 ± 0.20	34.9 ± 0.4	1.4 ± 0.2
Stem DCM	5.7 ± 0.6	40.8 ± 5.6	109.7 ± 3.6	11.5 ± 0.60	9.7 ± 2.1	2.9 ± 0.1
Leaf MeOH	34.7 ± 2.9	45.8 ± 0.9	395.3 ± 5.3	0.9 ± 0.10	39.0 ± 0.4	6.9 ± 0.4
Leaf DCM	5.0 ± 0.1	18.9 ± 0.7	310.4 ± 3.1	>20.0^a^	10.3 ± 2.1	20.6 ± 0.2

a Even at 20 mg mL^−1^ of extract only 17% of DPPH was inhibited.

### 3.3. Antimicrobial activity

All the tested *M.* × *giganteus* extracts exhibited antimicrobial activity, although the efficiency varied depending on the plant organ, extraction solvent, and the tested microorganism (Tables 3–5). MIC and MBC values ranged from 0.23 to 1.88 mg mL^−1^ and from 0.47 to 3.75 mg mL^−1^, respectively. Among the tested extracts, the MeOH rhizome and DCM stem extracts showed particularly strong antibacterial activity. The MeOH rhizome extract was most effective against *B. cereus* (MIC/MBC of 0.23/0.47 mg mL^−1^), while the DCM stem extract showed MIC values of 0.23–0.94 mg mL^−1^ across the tested bacteria, including MIC/MBC of 0.23/0.47 mg mL^−1^ against *B. cereus* as well. DCM stem extract also inhibited *S. aureus*, *L. monocytogenes*, *E. coli*, and *E. cloacae* at 0.47 mg mL^−1^. Contrary to this, MeOH root and leaf extracts and the DCM rhizome and leaf extracts generally showed slightly weaker antibacterial activity, with MIC values reaching 1.88 mg mL^−1^ for several microorganisms (Table 3). The results were comparable or superior to commercial preservatives E211 and E224. DCM extracts demonstrated lower MIC and MBC values than MeOH extracts.

**Table 3:** Antibacterial activity of *M.* × *giganteus* extracts (mg mL^−1^). MIC – minimum inhibitory concentration; MBC – minimum bactericidal concentration

		*S. aureus* (ATCC 11632)	*B. cereus* (clinical isolate)	*L. monocytogenes* (NCTC 7973)	*Salmonella enterica* serovar Typhi (ATCC 13311)	*E. coli* (ATCC 25922)	*E. cloacae* (ATCC 35030)
Root MeOH	MIC	1.88	0.94	1.88	0.94	1.88	1.88
	MBC	3.75	1.88	3.75	1.88	3.75	3.75
Root DCM	MIC	0.47	0.47	0.94	1.88	0.94	0.94
	MBC	0.94	0.94	1.88	3.75	1.88	1.88
Rhizome MeOH	MIC	0.47	0.23	0.94	0.94	0.94	0.94
	MBC	0.94	0.47	1.88	1.88	1.88	1.88
Rhizome DCM	MIC	1.88	0.94	1.88	0.94	1.88	1.88
	MBC	3.75	1.88	3.75	1.88	3.75	3.75
Stem MeOH	MIC	0.94	0.47	0.94	0.94	0.94	0.47
	MBC	1.88	0.94	1.88	1.88	1.88	0.94
Stem DCM	MIC	0.47	0.23	0.47	0.94	0.47	0.47
	MBC	0.94	0.47	0.94	1.88	0.94	0.94
Leaf MeOH	MIC	1.88	0.94	1.88	0.94	1.88	1.88
	MBC	3.75	1.88	3.75	1.88	3.75	3.75
Leaf DCM	MIC	1.88	0.94	0.94	1.88	1.88	1.88
	MBC	3.75	1.88	1.88	3.75	3.75	3.75
E211	MIC	4.00	0.50	1.00	1.00	1.00	2.00
	MBC	4.00	0.50	2.00	2.00	2.00	4.00
E224	MIC	1.00	2.00	0.5	1.00	0.50	0.50
	MBC	1.00	4.00	1.00	1.00	1.00	0.50

**Table 4:** Antifungal activity of *M.* × *giganteus* extracts (mg mL^−1^). MIC – minimum inhibitory concentration; MFC – minimum fungicidal concentration

		*A. fumigatus* (ATCC 9197)	*A. versicolor* (ATCC 11730)	*P. funiculosum* (ATCC 36839)	*P. verrucosum* var. *cyclopium* (food isolate)	*T. viride* (IAM 5061)
Root MeOH	MIC	0.47	0.23	0.23	0.47	0.47
	MFC	0.94	0.47	0.47	0.94	0.94
Root DCM	MIC	0.47	0.47	0.47	0.47	0.47
	MFC	0.94	0.94	0.94	0.94	0.94
Rhizome MeOH	MIC	0.47	0.47	0.47	0.94	0.47
	MFC	0.94	0.94	0.94	1.88	0.94
Rhizome DCM	MIC	0.94	0.94	0.47	0.47	0.47
	MFC	1.88	1.88	0.94	0.94	0.94
Stem MeOH	MIC	0.47	0.47	0.47	0.47	0.47
	MFC	0.94	0.94	0.94	0.94	0.94
Stem DCM	MIC	0.47	0.47	0.47	0.47	0.47
	MFC	0.94	0.94	0.94	0.94	0.94
Leaf MeOH	MIC	0.47	0.47	0.47	0.94	0.47
	MFC	0.94	0.94	0.94	1.88	0.94
Leaf DCM	MIC	0.94	0.94	0.94	0.94	0.94
	MFC	1.88	1.88	1.88	1.88	1.88
E211	MIC	1.00	2.00	1.00	2.00	1.00
	MFC	2.00	4.00	2.00	4.00	2.00
E224	MIC	1.00	1.00	0.50	1.00	0.50
	MFC	1.00	1.00	0.50	1.00	0.50

**Table 5:** Anticandidal activity of *M.* × *giganteus* extract (mg mL^−1^). MIC – minimum inhibitory concentration; MFC – minimum fungicidal concentration

		*C. albicans* 475/15	*C. albicans* 13/15	*C. albicans* 17/15	*C. parapsilosis* ATCC 22019	*C. tropicalis* ATCC 750	*C. krusei* H1/16
Root MeOH	MIC	0.50	1.00	1.00	0.50	0.25	0.50
	MFC	1.00	2.00	2.00	1.00	0.50	1.00
Root DCM	MIC	0.50	1.00	1.00	0.50	0.25	1.00
	MFC	1.00	2.00	2.00	1.00	0.50	2.00
Rhizome MeOH	MIC	0.50	1.00	0.50	1.00	0.50	1.00
	MFC	1.00	2.00	1.00	2.00	1.00	2.00
Rhizome DCM	MIC	1.00	0.50	0.25	1.00	0.50	0.25
	MFC	2.00	1.00	0.50	2.00	1.00	0.50
Stem MeOH	MIC	0.50	1.00	0.50	1.00	0.25	0.50
	MFC	1.00	2.00	1.00	2.00	0.50	1.00
Stem DCM	MIC	0.50	1.00	0.50	1.00	1.00	1.00
	MFC	1.00	2.00	1.00	2.00	2.00	2.00
Leaf MeOH	MIC	0.50	0.50	1.00	0.50	0.50	1.00
	MFC	1.00	1.00	2.00	1.00	1.00	2.00
Leaf DCM	MIC	0.50	1.00	1.00	0.50	0.25	1.00
	MFC	1.00	2.00	2.00	1.00	0.50	2.00
Ketoconazole (*x* × 10^−3^)	MIC	3.00	1.50	1.50	3.00	1.50	1.50
	MFC	6.00	51.00	51.00	6.00	6.00	3.00

Antifungal activity was quite consistent in all the tested extracts, with MIC values ranging from 0.23 to 0.94 mg mL^−1^ and MFC values from 0.47 to 1.88 mg mL^−1^ against the tested micromycetes. MeOH root extract showed the lowest MIC values against *A. versicolor* and *P. funiculosum* (0.23 mg mL^−1^), with corresponding MFC value in both cases of 0.47 mg mL^−1^. Both MeOH and DCM stem extracts also inhibited growth of all five tested fungi at 0.47 mg mL^−1^.

Anticandidal activity was observed at MIC values in range of 0.25–1.00 mg mL^−1^ and MFC values in range of 0.50–2.00 mg mL^−1^. *C. tropicalis* was most susceptible to the activity of tested extracts, with MIC values as low as 0.25 mg mL^−1^ for the MeOH and DCM root extracts, MeOH stem extract, and DCM leaf extract. The DCM rhizome extract also showed similar activity against *C. albicans* 17/15 and *C. krusei* (Table 5).

Most of the tested extracts showed better antifungal potential than E211 and E224 towards filamentous fungi, except for *P. funiculosum* and *T. viride*. *C. tropicalis* was the most susceptible *Candida* spp., particularly to MeOH stem and DCM leaf extracts, likely due to the high abundance of ellagic acid. Contrary to this, *C. albicans* 13/15 was the most resilient species. All extracts showed lower anticandidal activity than widely used antifungal agent ketoconazole.

### 3.4. Molecular docking

The docking analysis included *p*-coumaric acid, ellagic acid, 5-*O*-caffeoylquinic acid, and isoorientin as ligands, tested against key proteins crucial for disrupting bacterial and fungal survival mechanisms from: *A. fumigatus*, *B. cereus*, *C. albicans*, *E. coli*, *L. monocytogenes*, *S. enterica* serovar Typhi, *S. aureus*, and *C. tropicalis*. Among the tested complexes, isoorientin showed the highest binding affinity with *C. albicans*-XOG1 and *S. aureus*-MurE, highlighting its potential as a promising inhibitor against fungal and bacterial infections. The RMSD values ranged from 0.1–29.0, reflecting the structural stability and flexibility of the docked complexes. Compounds tested herein were selected for further investigation based on the following criteria: a binding affinity ≤−9 kcal mol^−1^, RMSD value ≤2, and ≥4 Hbonds (Fig. 3A and Table S2). Therefore, *p*-coumaric acid was not further analyzed. Five-*O*-caffeoylquinic acid demonstrated promising interactions with *L. monocytogenes*-PrfA and *B. cereus*-DltA, while ellagic acid showed high binding affinity with *C. albicans*-XOG1 and *S. enterica*-Gyrase B. The interactions of 5-*O*-caffeoylquinic acid, isoorientin, and ellagic acid, with *L. monocytogenes*-PrfA, *B. cereus*-DltA, and *S. enterica* serovar Typhi- Gyrase A/B, *C. albicans*-XOG1, and *S. aureus*-MurE proteins were evaluated by conducting 100 ns MD simulations followed by MM-PBSA analyses. The calculations focused on RMSD, RMSF, SASA, minimum binding distance, and H-bonds dynamics, allowing for a comprehensive analysis of the temporal structural changes in each complex, providing insights into the overall stability and binding properties of the ligand–protein interactions. The lowest mean RMSD value, hence more stable binding, was observed in the *C. albicans*-XOG1_isoorientin, while the highest mean RMSD value was found in the *L. monocytogenes*-PrfA_5-*O*-caffeoylquinic acid. The stability of the complexes was evaluated by dividing the simulation into time intervals and analyzing the trend of RMSD increase over time. The most stable complexes were *C. albicans*-XOG1_isoorientin and *C. albicans*-XOG1_ellagic acid, which exhibited low RMSD values with minimal fluctuations throughout the simulation. *S. enterica* serovar Typhi-Gyrase A_isoorientin, *B. cereus*-DltA_isoorientin, *L. monocytogenes*-PrfA_isoorientin, and *S. enterica*-Gyrase B_5-*O*-caffeoylquinic acid exhibited moderate stability. *L. monocytogenes*-PrfA_5-*O*-caffeoylquinic acid, *S. aureus*-MurE_isoorientin, and *B. cereus*-DltA_5-*O*-caffeoylquinic acid were identified as the least stable (Fig. 4A). *C. albicans*-XOG1_isoorientin and *C. albicans*-XOG1_ellagic acid demonstrated the highest stability in their binding regions (lowest RMSF), whereas the binding region of the *S. aureus*-MurE_isoorientin exhibited the greatest flexibility (highest RMSF) (Fig. 4B). The lowest SASA values were observed in *L. monocytogenes*-PrfA_5-*O*-caffeoylquinic acid and *L. monocytogenes*-PrfA_isoorientin, suggesting that these ligands are deeply positioned in the binding pocket and establish strong interactions with the protein core, whereas the *S. aureus*-MurE_isoorientin demonstrated the highest mean SASA value, suggesting that the ligand interacts with the protein surface, resulting in significant solvent exposure. The analysis of maximum SASA values showed that the *S. aureus*-MurE_isoorientin attained a maximum SASA value of 248.1 nm^2^, indicating substantial solvent contact and more exposed binding orientation. A similar trend was observed in the *B. cereus*-DltA_isoorientin and *B. cereus*-DltA_5-*O*-caffeoylquinic acid, which also exhibited elevated SASA values, suggesting ligand interactions occurring in closer proximity to the protein's outer surface. The temporal variations in SASA values revealed an increasing trend in specific complexes. For instance, in *L. monocytogenes*-PrfA_5-*O*-caffeoylquinic acid, *S. aureus*-MurE_isoorientin and *C. albicans*-XOG1_ellagic acid, the initial SASA value increased by the end of the 100 ns simulation, suggesting a gradual increase in ligand exposure. In *S. enterica*-Gyrase A_isoorientin and *C. albicans*-XOG1_isoorientin, SASA values remained consistent throughout the simulation, suggesting minimal conformational changes in the binding region (Fig. 4C). The analysis of average minimum distance indicated that the *S. enterica* serovar Typhi-GyraseA_isoorientin exhibited the lowest value, suggesting a highly stable and close interaction between the ligand and the protein. In contrast, the highest average minimum distance was observed in the *S. enterica*-GyraseB_5-*O*-caffeoylquinic acid, suggesting greater fluctuations during binding with periodic ligand displacement, though without complete dissociation. Moderate fluctuations were observed in the *L. monocytogenes*-PrfA_5-*O*-caffeoylquinic acid, while *B. cereus*-DltA_5-*O*-caffeoylquinic acid and *S. aureus*-MurE_isoorientin displayed minor variations, indicating relatively flexible interactions. The *B. cereus*-DltA_isoorientin and *L. monocytogenes*-PrfA_isoorientin remained close to the binding site, with the former showing particularly stable positioning. In the *C. albicans*-XOG1 complexes with ellagic acid or isoorientin, ligand positioning remained consistently stable. Overall, the results highlight a spectrum of binding behaviors, ranging from highly stable to more flexible interactions (Fig. 4D). A higher number of H-bonds indicates stronger ligand integration into the binding site, whereas a lower number suggests weaker or more transient binding. The highest average H-bonds values were observed in the *C. albicans*-XOG1_isoorientin and *B. cereus*-DltA_isoorientin (Fig. 5H and B); the lowest average H-bonds count was found in the *C. albicans*-XOG1_ellagic acid (Fig. 5E). *S. enterica*-GyraseA_isoorientin reached a notably high peak value (Fig. 5F), while *C. albicans*-XOG1_ellagic acid exhibited the lowest maximum, highlighting its relatively weak hydrogen bonding interactions (Fig. 5E). Temporal analysis showed dynamic interaction patterns. *L. monocytogenes*-PrfA_5-*O*-caffeoylquinic acid displayed a gradual increase in H-bond count, suggesting progressive strengthening of interactions during the simulation (Fig. 5G). In contrast, the *B. cereus*-DltA_isoorientin and *C. albicans*-XOG1_isoorientin, maintained high Hbonds counts but showed gradual declines, indicating slight weakening over time while remaining strongly bound (Fig. 5B and H). A significant decrease in H-bonds count was observed in *S. enterica* serovar Typhi-GyraseB_5-*O*-caffeoylquinic acid, indicating that binding interactions weakened over time (Fig. 5A). The *C. albicans*-XOG1_ellagic acid maintained a low H-bonds count but displayed an increasing trend (Fig. 5E). A pronounced decrease in H-bonds count was detected in *S. aureus*-MurE_isoorientin and *L. monocytogenes*-PrfA_isoorientin, indicating a substantial decline in binding stability (Fig. 5I and C). *B. cereus*-DltA_5-*O*-caffeoylquinic acid exhibited an increasing trend, suggesting a strengthening interaction within the binding region over time (Fig. 5D) unlike the *S. enterica*-GyraseA_isoorientin where gradual weakening of initial interactions occurred despite a high average H-bonds count (Fig. 5F).

**Fig. 2:**
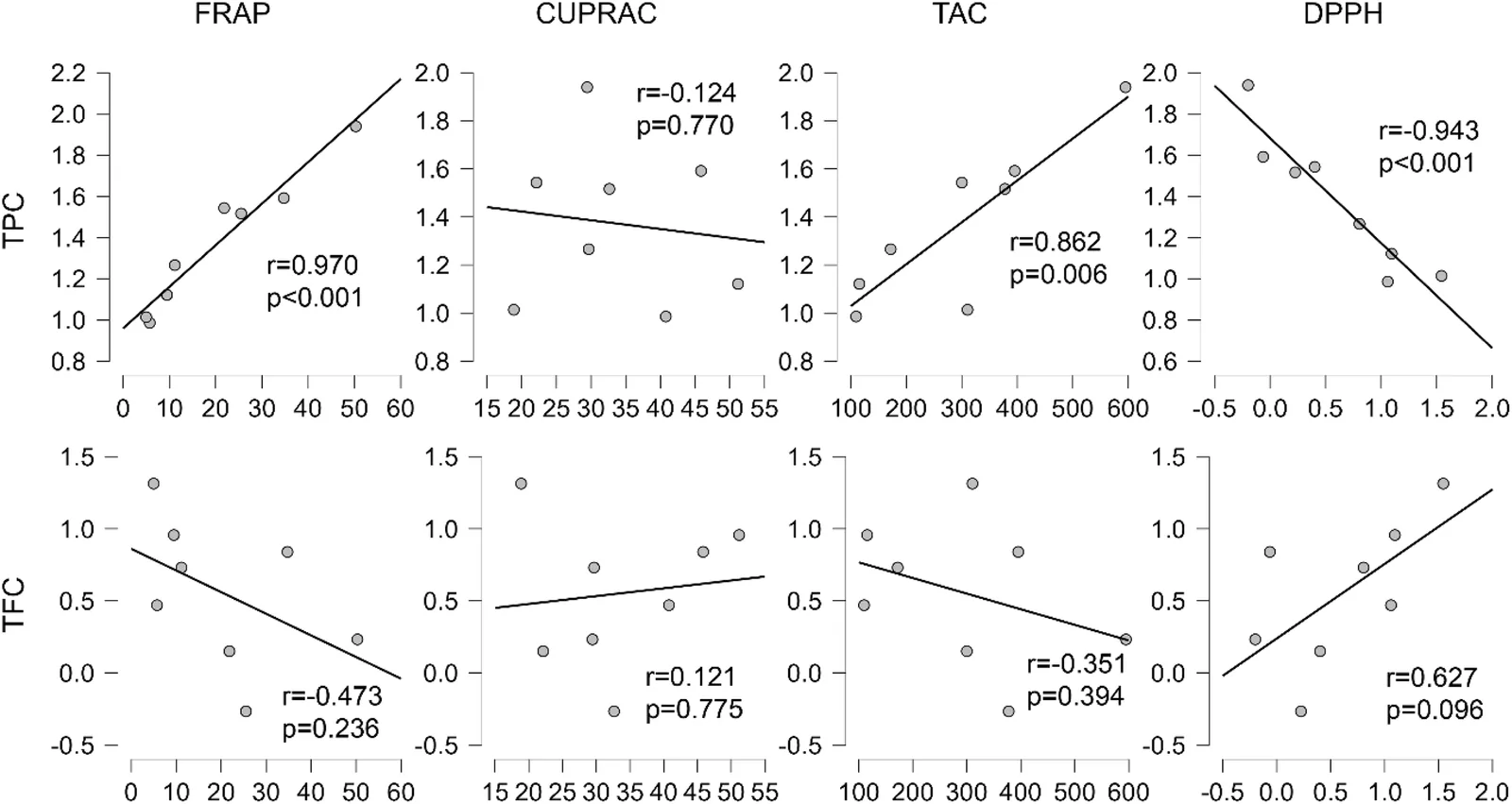
Pearson's correlation of total phenolic (TPC) and flavonoid content (TFC) with results of antioxidant assays. Pearson's r is given together with *p* value.

**Fig. 3:**
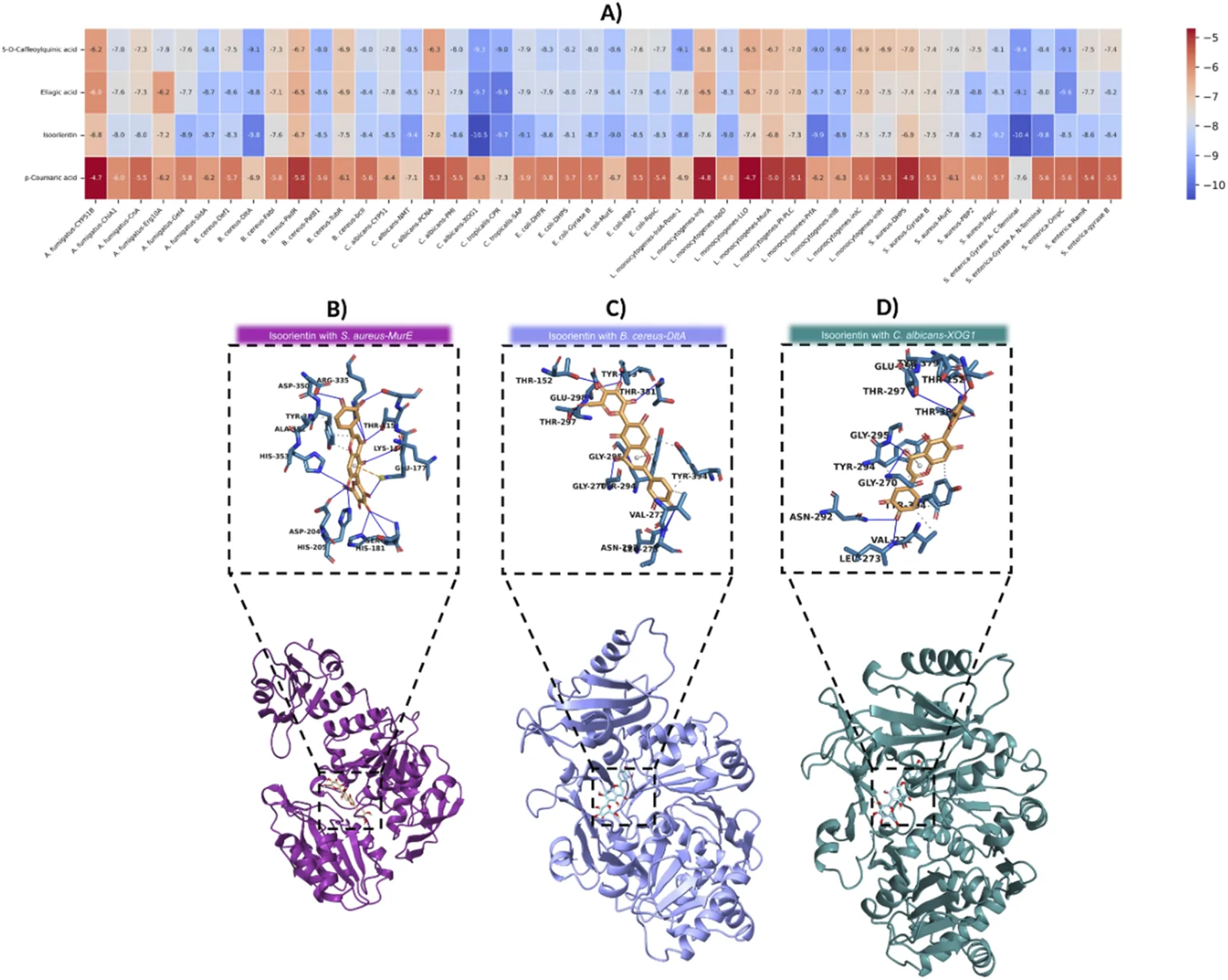
A comprehensive analysis of the binding interactions between enzymes/proteins and the selected compounds: (A) a graphical representation of docking scores for relevant enzymes/proteins. (B) Molecular interaction analysis of isoorientin with *S. aureus*-MurE. (C) Molecular interaction analysis of isoorientin with *B. cereus*-DltA. (D) Molecular interaction analysis of isoorientin with *C. albicans*-XOG.

**Fig. 4:**
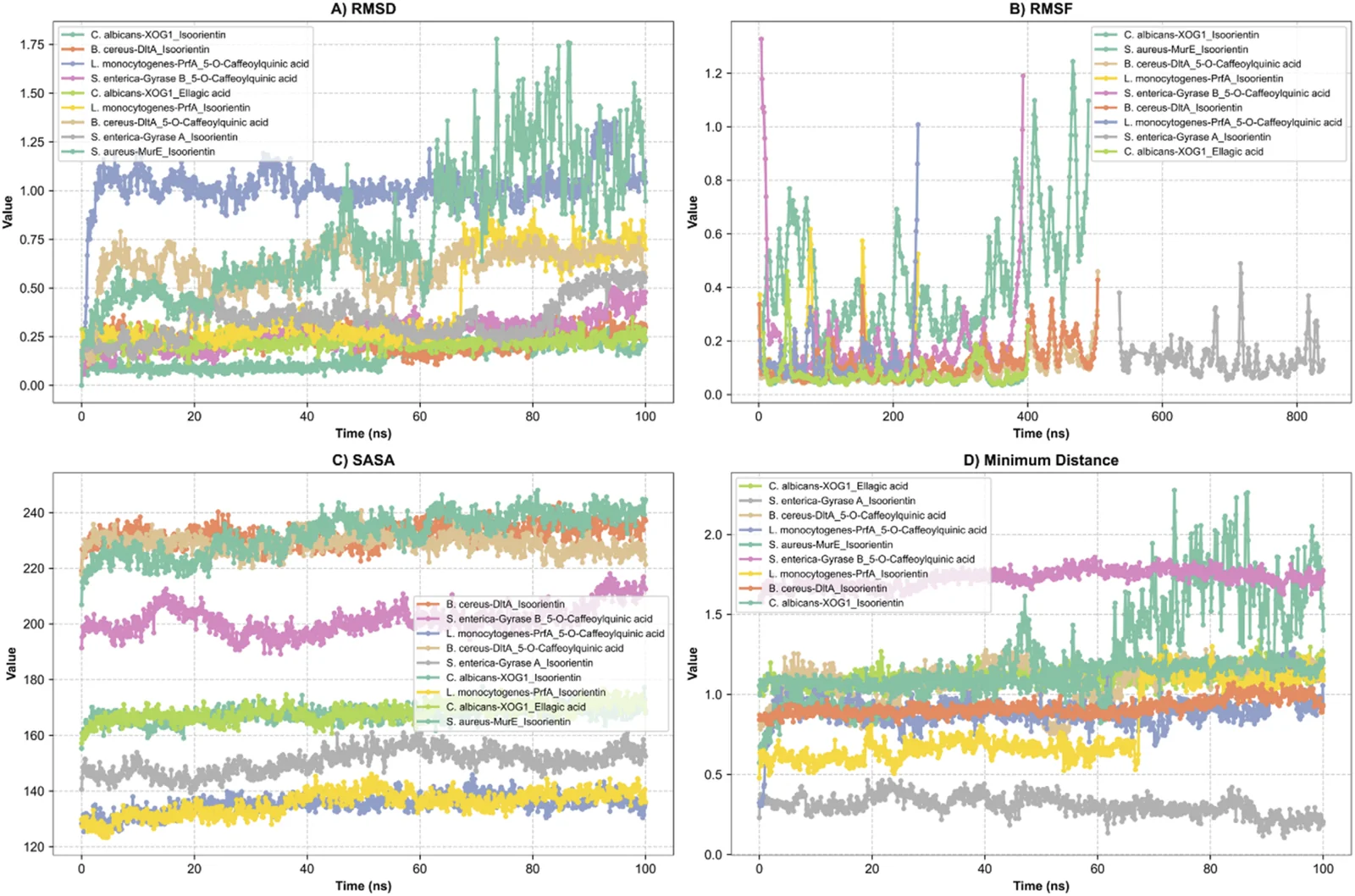
Presentation of molecular dynamics simulations in graphical form; (A) RMSD of the *S. enterica*-Gyrase B_5-*O*-caffeoylquinic acid, *B. cereus*-DltA_isoorientin, *L. monocytogenes*-PrfA_isoorientin, *B. cereus*-DltA_5-*O*-caffeoylquinic acid, *C. albicans*-XOG1_ellagic acid, *S. enterica*-GyraseA_isoorientin, *L. monocytogenes*-PrfA_5-*O*-caffeoylquinic acid, *C. albicans*-XOG1_isoorientin, and *S. aureus*-MurE_isoorientin complexes. (B) RMSF of the *S. enterica*-Gyrase B_5-*O*-caffeoylquinic acid, *B. cereus*-DltA_isoorientin, *L. monocytogenes*-PrfA_isoorientin, *B. cereus*-DltA_5-*O*-caffeoylquinic acid, *C. albicans*-XOG1_ellagic acid, *S. enterica*-GyraseA_isoorientin, *L. monocytogenes*-PrfA_5-*O*-caffeoylquinic acid, *C. albicans*-XOG1_isoorientin, and *S. aureus*-MurE_isoorientin complexes. (C) Solvent accessibility of the *S. enterica*-Gyrase B_5-*O*-caffeoylquinic acid, *B. cereus*-DltA_isoorientin, *L. monocytogenes*-PrfA_isoorientin, *B. cereus*-DltA_5-*O*-caffeoylquinic acid, *C. albicans*-XOG1_ellagic acid, *S. enterica*-GyraseA_isoorientin, *L. monocytogenes*-PrfA_5-*O*-caffeoylquinic acid, *C. albicans*-XOG1_isoorientin, and *S. aureus*-MurE_isoorientin complexes. (D) Minimum distance of the *S. enterica*-Gyrase B_5-*O*-caffeoylquinic acid, *B. cereus*-DltA_isoorientin, *L. monocytogenes*-PrfA_isoorientin, *B. cereus*-DltA_5-*O*-caffeoylquinic acid, *C. albicans*-XOG1_ellagic acid, *S. enterica*-GyraseA_isoorientin, *L. monocytogenes*-PrfA_5-*O*-caffeoylquinic acid, *C. albicans*-XOG1_isoorientin, and *S. aureus*-MurE_isoorientin complexes.

**Fig. 5:**
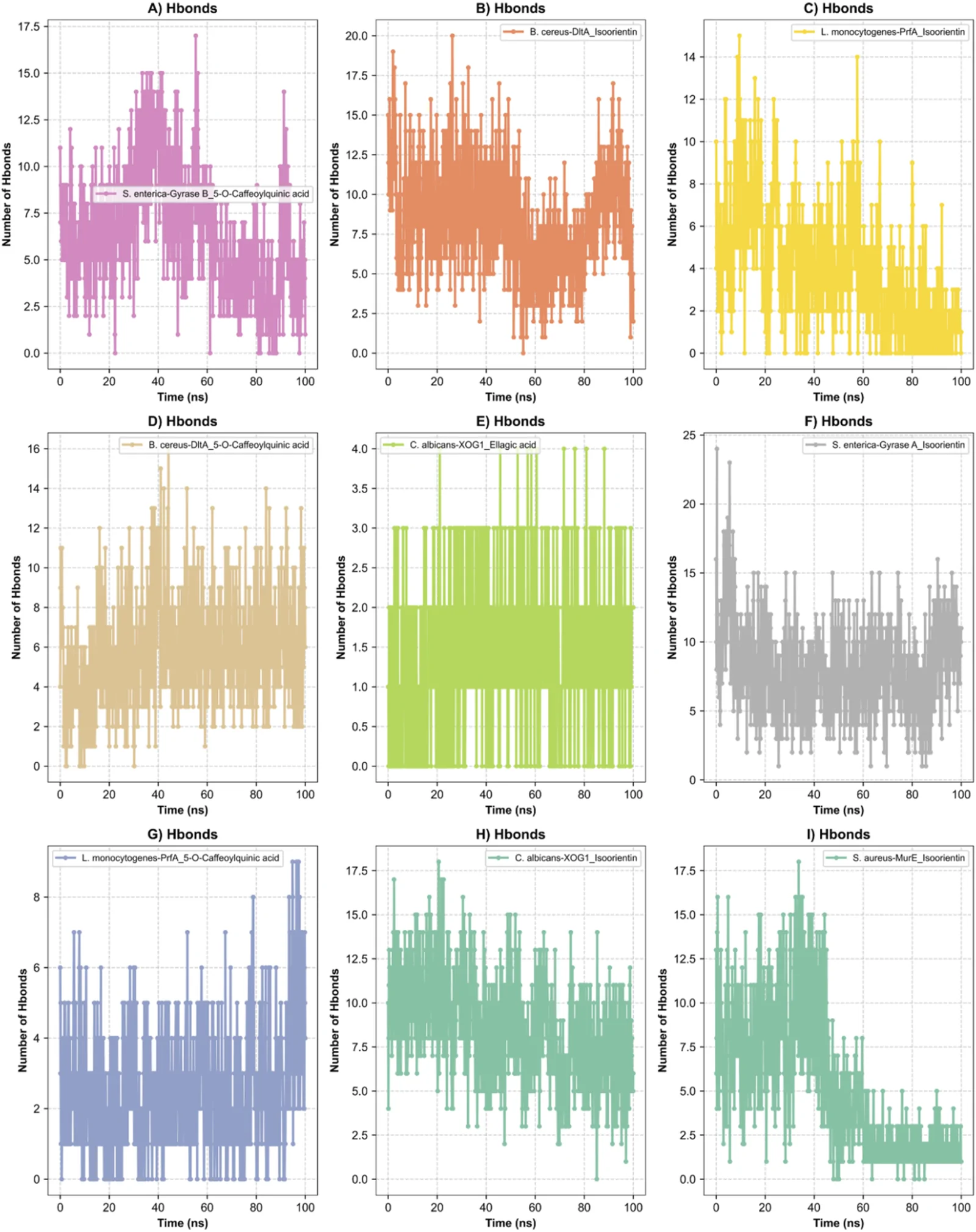
Hydrogen bonds analysis. (A) Hydrogen bonds of *S. enterica*-Gyrase B_5-*O*-caffeoylquinic acid complex. (B) Hydrogen bonds of the *B. cereus*-DltA_isoorientin complex. (C) Hydrogen bonds of the *L. monocytogenes*-PrfA_isoorientin complex. (D) Hydrogen bonds of the *B. cereus*-DltA_5-*O*-caffeoylquinic acid complex. (E) Hydrogen bonds of the *C. albicans*-XOG1_ellagic acid complex. (F) Hydrogen bonds of the *S. enterica*-GyraseA_isoorientin complex. (G) Hydrogen bonds of the *L. monocytogenes*-PrfA_5-*O*-caffeoylquinic acid. (H) Hydrogen bonds of the *C. albicans*-XOG1_isoorientin. (I) Hydrogen bonds of the *S. aureus*-MurE_isoorientin.

**Fig. 6:**
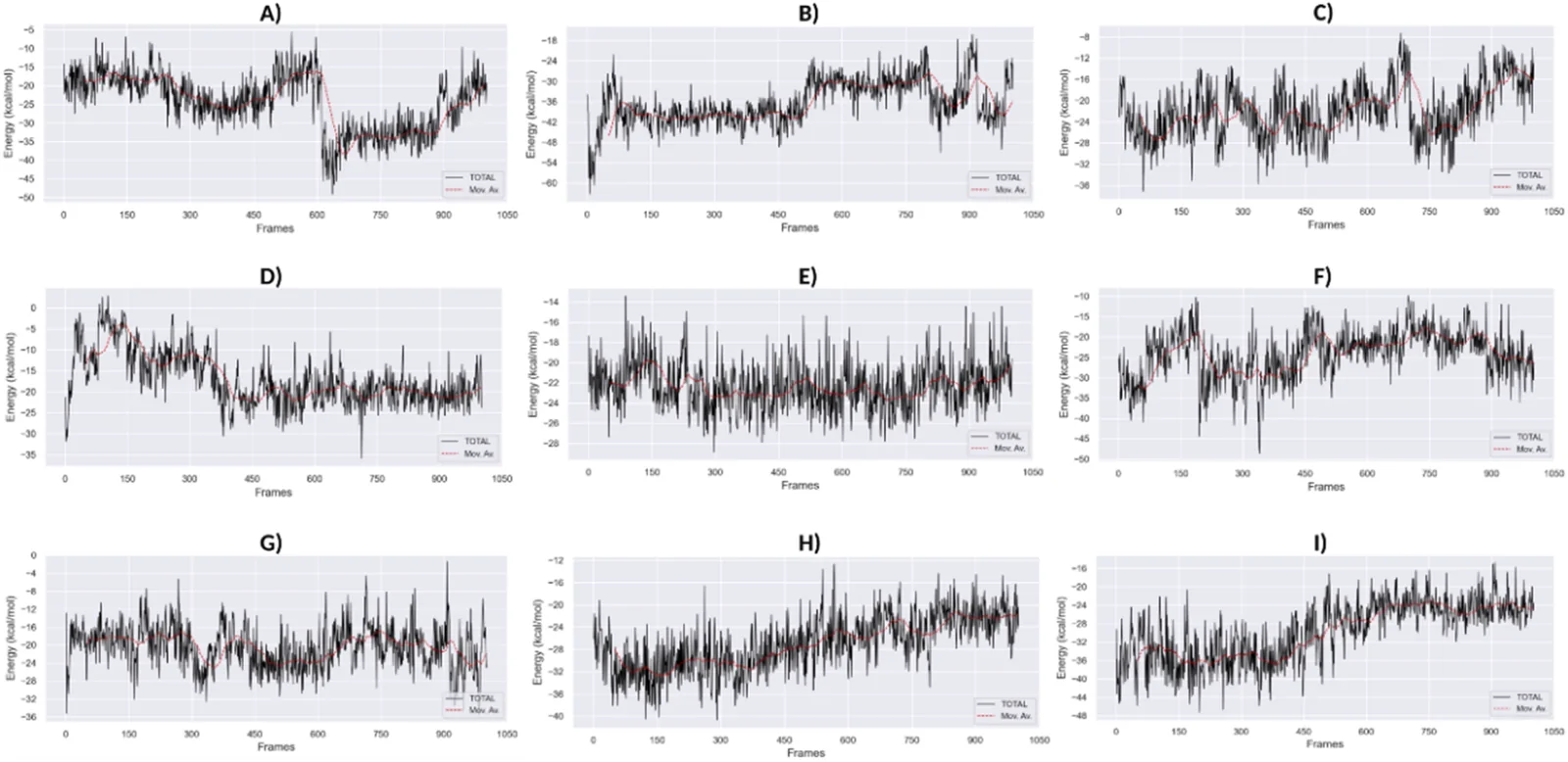
MM-PBSA analysis. (A) *S. enterica*-Gyrase B_5-*O*-caffeoylquinic acid complex. (B) *B. cereus*-DltA_isoorientin complex. (C) *L. monocytogenes*-PrfA_isoorientin complex. (D) *B. cereus*-DltA_5-*O*-caffeoylquinic acid complex. (E) *C. albicans*-XOG1_ellagic acid complex. (F) *S. enterica*-GyraseA_isoorientin complex. (G) *L. monocytogenes*-PrfA_5-*O*-caffeoylquinic acid. (H) *C. albicans*-XOG1_isoorientin. (I) *S. aureus*-MurE_isoorientin.

The stability of the complexes was assessed by considering the temporal trends in energy fluctuations across 1001 frames over a 100 ns simulation. *S. enterica*-GyraseA_isoorientin and *C. albicans*-XOG1_isoorientin emerged as the most stable complexes, as their energy levels quickly plateaued with minimal fluctuations. *S. enterica* serovar Typhi-GyraseB_5-*O*-caffeoylquinic acid now revealed moderate stability. Conversely, *B. cereus*-DltA_5-*O*-caffeoylquinic acid, *S. aureus*-MurE_isoorientin, and *L. monocytogenes*-PrfA_5-*O*-caffeoylquinic acid exhibited stabilization to a certain extent, though they continued to demonstrate minor fluctuations. The complexes that demonstrated the least stability were identified as *L. monocytogenes*-PrfA_isoorientin and *C. albicans*-XOG1_ellagic acid, where the energy levels fluctuated continuously without forming a clear plateau (Fig. 6A–I). When considering the collective contributions of RMSD, RMSF, SASA, minimum binding distance, H-bonds, and MM-PBSA energy analyses, *S. enterica*-Gyrase B_5-*O*-caffeoylquinic acid and *S. aureus*-MurE_isoorientin display moderate or low binding stability, indicating the need for more extensive simulations or experimental validation. Conversely, ellagic acid demonstrates noteworthy activity against *C. albicans*-XOG1, while isoorientin exhibits notably high stability and robust binding characteristics in complexes with *C. albicans*-XOG1, *S. enterica*-Gyrase A, and *B. cereus*-DltA, suggesting its potential as a promising inhibitor candidate.

### 3.5. *In situ* activity of leaf MeOH extract in milk

Based on the results we obtained *in vitro* and *in silico*, we evaluated whether the antimicrobial activity could be retained *in situ* using commercial sample of milk as a matrix for bacterial growth. At 24 h, the solvent control caused 34% inhibition, while the methanolic leaf extract demonstrated substantially higher activity, reaching 95% and 97% inhibition at 1 and 2 mg mL^−1^, respectively (Table 6). After 48 h, inhibition decreased to 25% in the solvent control and to 28% at 1 mg mL^−1^, whereas the extract at 2 mg mL^−1^ stayed quite active with 81% inhibition.

**Table 6:** Growth inhibition of *B. cereus* in milk (%) given as mean with standard deviation

	Inhibition 24 h	Inhibition 48 h
Control solvent	34 ± 6	25 ± 4
Extract 1 mg mL	95 ± 1	28 ± 4
Extract 2 mg mL	97 ± 1	81 ± 5

## 4 Discussion

### 4.1. Chemical analysis

The high abundance of *p*-coumaric acid in roots observed in this study may reflect its involvement in root microbe interactions, antioxidant defense, and rhizosphere signaling.^9^ The decreasing trend in bioactive compound content from roots to aerial tissues is consistent with previous findings reported for *Miscanthus* species.^10^ Solvent selection strongly influenced extraction efficiency: MeOH preferentially recovered polar phenolic acids and flavonoid glycosides, whereas DCM generally extracted lower amounts of these compounds. Nevertheless, quercetin-3-*O*-glucoside and ellagic acid were more abundant in certain DCM extracts, indicating that DCM can selectively recover specific flavonoids despite its lower overall extraction efficiency. The phytochemical profile of *M.* × *giganteus* largely resembles that of its parental species. Like *M. sacchariflorus*, it contains chlorogenic acid, *p*-coumaric acid, vitexin, and luteolin, although ferulic and *p*-hydroxybenzoic acids have also been reported in the parent species.^11^ Likewise, *M. sinensis* is characterized by abundant *O*-cinnamoylquinic acids and flavones, including glycosylated derivatives.^12^ Comparative analyses further confirmed that *M. sinensis*, *M. sacchariflorus*, and *M.* × *giganteus* share a largely conserved phenolic acid profile comprising *p*-hydroxybenzoic, isovanillic, syringic, *p*-coumaric, ferulic, and isoferulic acids, together with *trans*-cinnamic acid derivatives.^2^

### 4.2. Antioxidant activity

Tested samples were particularly rich in *p*-coumaric acid, 3-*O*-caffeoylquinic acid and 5-*O*-caffeoylquinic acid, well-documented antioxidants.^13,14^ These compounds contributed to the overall good antioxidant activity of extracts, with the highest observed in the root MeOH extract. Results we obtained regarding antioxidant potential of *M.* × *giganteus* align with previous studies.^15^*M.* × *giganteus* lignins exhibited strong antioxidant activity in ABTS, DPPH, and β-carotene bleaching assays,^16^ with DPPH EC_50_ values ranging from 7.6–29.0 µg mL^−1^, and ABTS values 64.1–154.6 mg Trolox/g extract. Similarly, *M. sinensis* extracts demonstrated considerable antioxidant activity.^17^

Given the exceptionally high concentration of *p*-coumaric acid in MeOH root extracts (22 511.64 µg g^−1^), it was expected that this extract would exhibit the highest antioxidant activity in FRAP assay reflecting reducing power of phenolic acids. Our further analysis did show strong positive correlations between TPC with the results of FRAP and TAC, and negative with the results of DPPH assay (*p* < 0.05). Similarly, MeOH leaf extract also demonstrated high reducing potential (34.7 mg AAE g^−1^), likely due to high levels of chlorogenic or 3-*O*-caffeoylquinic acid and 5-*O*-caffeoylquinic acid. Overall, these findings support the major contribution of phenolic compounds to the observed antioxidant activity.

The negative correlation with DPPH values was expected, as lower extract concentrations required to quench DPPH radical indicate its stronger scavenging activity (Fig. 2). Correlation values obtained for flavonoids imply they do not contribute to the overall extracts' effects. The content of phenolic compounds was 3.7 times higher in MeOH compared to DCM extracts, whereas the opposite was observed for flavonoids. Higher values of MeOH extracts in TAC (except in case of leaf) and FRAP, as well as lower values obtained in DPPH assay, can be attributed to overall higher phenolics content in them. Results of CUPRAC assay indicate similar contribution of both groups of metabolites. Differences in FRAP and CUPRAC results originate in the assay nature, as CUPRAC can measure both lipophilic, hydrophilic and sulfur-containing antioxidants.^18^ Observed variability in various assays suggest the diverse antioxidant mechanisms present in *M.* × *giganteus* extracts. The interactions between phenolic acids and flavonoids likely enhance the overall antioxidant capacity through synergistic effects, as previously demonstrated.^15^

### 4.3. Antimicrobial activity

The observed antimicrobial activity may at least partly be associated with the phenolic constituents identified in the extracts. *p*-Coumaric acid, which can impair bacterial DNA integrity,^19^ was particularly abundant in the MeOH root, rhizome, and stem extracts and may contribute to their antibacterial effects. Ellagic acid, detected in all extracts in moderate amounts, has been previously associated with antifungal activity through membrane disruption and inhibition of key enzymatic processes.^20^ Overall, phenolic acids and flavonoids are known to exert antimicrobial activity through multiple mechanisms, including alterations in membrane permeability, enzyme inhibition, and interference with microbial metabolism, which may have contributed to the antimicrobial activity observed in the present study.^21^ Our findings are consistent with previous reports demonstrating the antibacterial activity of *Miscanthus* extracts against microorganisms such as *S. aureus* and *E. coli*,^16^ with similar antimicrobial effects also reported for supercritical fluid extracts of *Miscanthus* species.^2^

*Miscanthus*, having considerable lignin content, represents a promising source for the production of lignin-based packaging materials, especially since lignocellulosic biomass is increasingly recognized as a valuable source of materials for the development of sustainable food-packaging systems. Incorporation of lignin into biopolymers such as polylactic acid, chitosan, or starch-based materials has been shown to improve mechanical, UV-protective, and bioactive properties.^22^ The antimicrobial activity of *M.* × *giganteus* extracts shown herein provides an additional functional advantage and further supports the potential valorization of this biomass in active food-packaging applications. Although some challenges remain, lignin-based active packaging represents a promising strategy for extending food shelf life and reducing spoilage while contributing to circular bioeconomy principles.^23^

### 4.4. Molecular docking

Molecular docking simulations were performed using hub molecules derived from *M.* × *giganteus* to evaluate their binding affinity toward selected bacterial and fungal target proteins. The proteins were chosen based on their relevance to pathogenicity, antimicrobial resistance, or essential cellular processes, as well as the availability of high-resolution structural data to ensure reliable docking performance. This strategy is consistent with previous *in silico* studies aimed at identifying potential antimicrobial or antifungal inhibitors.^19^ The analyses showed that several *M.* × *giganteus* metabolites, particularly isoorientin, ellagic acid, and 5-*O*-caffeoylquinic acid, can strongly interact with key bacterial and fungal target proteins involved in pathogenicity and essential cellular functions. Among all tested complexes, isoorientin consistently demonstrated the most stable binding, especially with *C. albicans* XOG1, *S. enterica* serovar Typhi Gyrase A, and *B. cereus* DltA, as supported by multiple structural stability parameters. Ellagic acid also showed strong and stable interactions, particularly against *C. albicans* XOG1, while 5-*O*-caffeoylquinic acid exhibited moderate but sometimes less stable binding depending on the target protein. Overall, the simulation results suggest that these compounds may interfere with microbial survival mechanisms. Based on the *in silico* results, the leaf MeOH extract was selected for *in situ* evaluation against *B. cereus* due to its high content in isoorientin and 5-*O*-caffeoylquinic acid, which demonstrated the most stable and favorable binding interactions with key bacterial target proteins.

### 4.5. *In situ* activity of leaf MeOH extract in milk

*B. cereus* is widespread in the environment and can form spores and biofilm, thus surviving harsh environmental conditions such as heating, freezing, drying and UV radiation.^20^ The strains produce toxins responsible for food intoxication and infection, including cereulide, as well as hemolysin BL and cytotoxin K, which are more susceptible to various treatments.^21^ In addition, rancidity and bitterness of milk and dairy products are attributed to the presence of *B. cereus* strains. Notably, *B. cereus* shows enhanced growth in pasteurized milk compared to the raw sample, likely due to conditions that favor the germination of spores embedded in biofilms, which can consist of single or multiple species. Given that the widespread occurrence of *B. cereus* is mainly driven by contamination, controlling bacterial toxins in food products requires addressing the root cause, which is the elimination of *B. cereus* itself, as recently recommended by the European Food Safety Authority.^22^

Results we obtained indicate that *in situ* effect of the methanolic leaf extract towards *B. cereus* was dose dependent, with the higher concentration maintaining inhibitory activity even after prolonged incubation. Retention of this high inhibitory activity after 48 h is particularly important since it suggests that the tested extract remains active under realistic food related conditions, as well as accelerated spoilage conditions. In addition to microbial contamination, oxidative degradation can also contribute to milk spoilage and negatively affect its organoleptic properties.^23^ Since our extract also demonstrated *in vitro* antioxidant activity, it may offer a potential dual beneficial strategy for milk preservation, simultaneously inhibiting bacterial growth and mitigating oxidative deterioration. Such behavior increases the value of the extract and supports its potential as a natural preservative candidate in the dairy industry. This is important because prolonged suppression of *B. cereus* in dairy systems is more relevant for shelf-life extension and contamination control than short-term inhibition alone. Moreover, this puts *M.* × *giganteus* on a list with few other plants that could be incorporated as natural food preservatives.^24^

## 5 Conclusion

This study highlights *M.* × *giganteus* as a rich natural source of bioactives with significant antioxidant and antimicrobial properties. Among the compounds identified, *p*-coumaric acid was the most abundant, particularly in MeOH root extracts, followed by significant amount of isoorientin and ellagic acid. Solvent polarity played a key role in extraction efficiency, with MeOH proving more efficient than DCM for most phenolics. The extracts exhibited strong antioxidant activity in all assays, whereas antimicrobial assays revealed promising activity towards pathogenic microorganisms, in some cases surpassing commercial preservatives. Molecular docking confirmed high binding affinities of selected compounds to microbial target proteins. *In situ* assays conducted in milk verified the inhibitory activity against *B. cereus*, reinforcing the promise of *M.* × *giganteus* as a natural source of antioxidants and antimicrobial compounds primarily for food applications. Future research should focus on thorough characterization and assessment of the cost effectiveness of production.

## Author contributions

DR: conceptualization, methodology, validation, formal analysis, investigation, writing, supervision. DS: investigation, writing. UG: methodology, formal analysis, investigation, writing. GZ: methodology, software, formal analysis, investigation, writing, visualization. MVC: methodology, software, formal analysis, investigation. JP: conceptualization, methodology, validation, formal analysis, investigation, writing, supervision.

## Conflicts of interest

The authors declare no competing interests.

## Abbreviations

### Biological, Chemical and Microbiological

MeOH: Methanol
DCM: Dichloromethane
E211: Sodium benzoate
E224: Potassium metabisulfite
DNA: Deoxyribonucleic acid
XOG1: Exo-β-1,3-glucanase 1
DltA: d-Alanine–d-alanyl carrier protein ligase
PrfA: Positive regulatory factor A
TAC: Total antioxidant capacity
CUPRAC: Cupric ion reducing antioxidant capacity
FRAP: Ferric reducing antioxidant power
TPC: Total phenolic content
TFC: Total flavonoid content
ATCC: American type culture collection
NCTC: National collection of type cultures
MBC: Minimum bactericidal concentration
MFC: Minimum fungicidal concentration

### Instrumental techniques

HESI: Heated electrospray ionization
RMSD: Root mean square deviation
RMSF: Root mean square fluctuation
SASA: Solvent accessible surface area
MM-PBSA: Molecular mechanics Poisson–Boltzmann surface area

## Supplementary Material

RA-OLF-D6RA07636J-s001

## Data Availability

Data will be requested from authors. Supplementary information (SI): Table S1: retention time, molecular ion and major MS^2^ fragments with collision energies of all tested compounds. Table S2: the docking score (kcal mol^−1^) and interacting residues of the enzyme and protein. See DOI: https://doi.org/10.1039/d6ra07636j.
