# Supplementary material for: Antimicrobial potential of Miscanthus × giganteus extracts: linking in vitro and in silico activity with in situ efficacy in milk
Source: RSC Adv. 2026 Sep 25. Online ahead of print. doi: 10.1039/d6ra07636j (PMC13613685; doi:10.1039/d6ra07636j)
Supplement: Supplementary file 1 [file RA-OLF-D6RA07636J-s001.pdf]

Supplementary Information (SI) for RSC Advances.
This journal is © The Royal Society of Chemistry

Molecular modelling

Protein and ligand preparation

Molecular docking analysis was carried out to gain insights into the interaction of the major phytochemicals in the extract of *M.* x *giganteus*. All 3D structures of selected proteins, identified using the uniprot platform (https://www.uniprot.org/), were downloaded from the Protein Data Bank (PDB) (https://www.rcsb.org/) by searching with their respective PDB IDs.

A. fumigatus: ChiA1 (PDB ID: 4TX6) (Lockhart et al., 2014), CYP51B (PDB ID: 4UYM) (Lockhart et al., 2014), Erg10A (PDB ID: 6L2C) (Zhang et al., 2020), Gel4 (PDB ID: 8PE1) (Redrado-Hernández et al., 2024), CnA (PDB ID: 6TZ7) (Juvvadi et al., 2019), SidA (PDB ID: 4B63) (Franceschini et al., 2012).

B. cereus: Def1 (PDB ID: 1WS0) (Moon et al., 2005), DltA (PDB ID: 3FCE) (Osman et al., 2009), FabI (PDB ID: 5YCX) (Kim et al., 2017), bcII (PDB ID: 1BVT) (Carfi et al., 1998), PadR (PDB ID: 4ESF) (Fibriansah et al., 2012), PatB1 (PDB ID: 5V8D) (Sychantha et al., 2018), TubR (PDB ID: 6AHT) (Hayashi et al., 2018).

C. albicans: XOG1 (PDB ID: 2PBO), NMT (PDB ID: 1IYK) (Sogabe et al., 2002), PMI (PDB ID: 1PMI) (Cleasby et al., 1996), PCNA (PDB ID: 7BUP) (Sundaram et al., 2021), CYP51 (PDB ID: 5TZ1) (Hargrove et al., 2017).

C. tropicalis: SAP (PDB ID: 1J71) (Symersky et al., 1997), CPR (PDB ID: 6T1U) (Ebrecht et al., 2019). ***E. coli***: DHFR (PDB ID: 1RX2) (Sawaya and Kraut, 1997), DHPS (PDB ID: 5V7A) (Dennis et al., 2018), Gyrase B (PDB ID: 1KZN) (Lafitte et al., 2002), MurE (PDB ID: 1E8C) (Gordon et al., 2001), YcbB (PDB ID: 6NTW) (Caveney et al., 2019), RpsC (PDB ID: 4V53) (Borovinskaya et al., 2007).

L. monocytogenes: InlA (PDB ID: 1O6T) (Schubert et al., 2002), PI-PLC (PDB ID: 1AOD) (Moser et al., 1997), PrfA (PDB ID: 6EXL) (Kulén et al., 2018), InlB (PDB ID: 4J4L), InlC (PDB ID: 1XEU) (Ooi et al., 2006), InlJ (PDB ID: 3BZ5) (Bublitz et al., 2008), LLO

(PDB ID: 4CDB) (Köster et al., 2014), InlH (PDB ID: 1H62) (Barna et al., 2001), MurA (PDB ID: 3R38), IspD (PDB ID: 3F1C).

S. aureus: DHPS (PDB ID: 1AD4) (Hampele et al., 1997), ParE similar to Gyrase B (PDB ID: 4URN) (Lu et al., 2014), PBP4 (PDB ID: 5TW8) (Alexander et al., 2018), RpsC (PDB ID: 5TCU) (Belousoff et al., 2017), MurE (PDB ID: 4C13) (Wang et al., 2021).

***S. enterica* serovar Typhi**: Gyrase A (C-Terminal) (PDB ID: 5ZTJ) (Sachdeva et al., 2020), Gyrase A (N-Terminal) (PDB ID: 8J9T), Gyrase B (PDB ID: 5ZXM) (Gupta et al., 2021), OmpC (PDB ID: 3UU2), RamR (PDB ID: 6IE9) (Yamasaki et al., 2019). Furthermore, all 3D structures of ligands were downloaded from the PubChem database (https://pubchem.ncbi.nlm.nih.gov/) and optimized using Avogadro v1.2.0. All ligands and proteins were prepared for docking using AutoDock v4.2.6 (Trott and Olson, 2010).

Docking grid and parameters

The docking grid files were generated either from the literature or using POCASA V1.1 (https://g6altair.sci.hokudai.ac.jp/g6/service/pocasa/).

A. fumigatus: ChiA1 (12.924, 36.379, 79.997; 40×40×126 Å), Erg10A (-12.406, -10.696, 22.028; 40×40×86 Å), CYP51B (137.837, 137.837, -1.651; 80×96×66 Å), Gel4 (-54.185, 100.228, -33.691; 34×32×28 Å), CnA (-28.185, -40.293, -71.911; 26×26×26 Å), SidA (-21.008, -29.176, 7.258; 94×68×112 Å).

B. cereus: Def1 (-33.17, 1.376, 78.664; 40×40×40 Å), DltA (24.194, -3.366, 17.828; 76×48×78 Å), Fabl (-14.603, -2.184, 19.066; 40×40×40 Å), bcII (8.11, 33.514, 46.50; 64×64×64 Å), PadR (3.81, 18.166, -4.846; 84×62×56 Å), PatB1 (237.171, -4.531, 113.48; 84×96×118 Å), TubR (6.438, 55.563, 12.173; 68×80×50 Å).

C. albicans: XOG1 (34.165, 41.086, 59.802; 40×40×60 Å), NMT (14.687, 45.48, 39.958; 80×70×70 Å), PMI (12.225, 11.504, 49.679; 40×40×40 Å), PCNA (-37.56, -8.006, -19.558; 50×48×74 Å), CYP51 (62.587, 66.769, 5.893; 110×94×114 Å).

C. tropicalis: SAP (30.033, 16.188, 15.817; 48×62×52 Å), CPR (-16.885, -1.815, -55.653; 126×126×126 Å). ***E. coli***: DHFR (27.438, 51.345, 13.311; 94×68×86 Å), DHPS (-17.836, -17.836, 103.740; 60×60×60 Å), Gyrase B (12.467, 27.336, 44.916; 60×60×60 Å), MurE (45.098, 37.112, 76.674; 70×60×70 Å), YcbB (16.929, -32.370, 42.151; 60×60×60 Å), RpsC (130.966, 32.099, 0.385; 88×88×88 Å).

L. monocytogenes: PI-PLC (30.236, 37.512, 21.541; 64×94×108 Å), PrfA (-17.276, -14.527, 9.533; 40×40×40 Å), InlA-Pose-1 (-7.839, 15.509, 64.698; 88×70×50 Å), InlA-Pose-2 (-5.921, -11.917, 4.481; 88×70×50 Å), inlB (-11.043, 4.967, -23.943; 62×46×78 Å), inlC (23.703, 23.065, 15.047; 50×40×26 Å), InlJ (79.807, -12.073, -97.841; 40×40×40 Å), LLO (-6.104, 22.138, -52.133; 100×76×100 Å), inlH (2.075, 9.185, 37.067; 76×76×76 Å), MurA (18.169, 38.408, 28.905; 54×88×66 Å), IspD (-16.63, 18.069, -39.052; 90×40×94 Å).

***S. enterica* serovar Typhi**: Gyrase A-C-Terminal (24.448, 23.03, 24.901; 46×44×48 Å), Gyrase A-N-Terminal (12.798, 69.609, -28.236; 40×40×40 Å), Gyrase B (-9.464, -8.996, 28.863; 94×50×78 Å), OmpC (-22.218, -38.38, 30.001; 104×70×68 Å), RamR (-12.186, -30.985, 10.385; 60×84×64 Å).

S. aureus: DHPS (32.46, 6.683, 42.972; 60×60×60 Å), Gyrase B (31.684, 5.252, 1.572; 60 X 60 X 40 Å), PBP4 (21.390, -62.210, 39.196; 60×60×60 Å), RpsC (99.46, 230.082, 201.387; 76×76×76 Å), MurE (-23.122, 2.508, 9.873; 60×60×60 Å).

Validation and interaction analysis

The grid box dimensions were specified as per the respective protein-ligand binding sites. AutoDock Vina V1.1.2 (https://autodock.scripts.edu) (Morris et al., 2009) was used to search for ligand distinct conformations, with the exhaustiveness set to 32 (Trott and Olson, 2010). To validate the accuracy of the docking process, the proteins were re-docked with their co-crystallized ligands, and RMSD values were calculated. To gain deeper insight into the protein/enzyme-ligand interactions, we employed the Protein-Ligand Interaction Profiler

(PLIP) (https://plip-tool.biotec.tu-dresden.de/plip-web/plip/index), which highlighted critical interactions, particularly hydrogen bonds (Adasme et al., 2021).

Molecular Dynamics Simulations and MM/PBSA Free Energy Calculation

Molecular dynamics (MD) simulations were performed using the CHARMM GUI platform (https://charmm-gui.org) (Jo et al., 2008). The System preparation followed the established guidelines of the Solution Builder tool (Jo et al., 2008). Protein parameterization utilized the CHARMM36m force field, aligning with previously described methodologies (Maier et al., 2015). A periodic boundary box contained TIP3P water molecules to ensure at least 10 Å separation between the protein and the box edges. Counterions were included to maintain electroneutrality and to achieve a NaCl concentration of 0.15 M. Management of electrostatic and van der Waals interactions relied on the Verlet cutoff scheme, while bond length constraints were enforced by the Linear Constraint Solver (LINCS) algorithm. Long-range electrostatic calculations were performed using the Particle Mesh Ewald (PME) method. Energy minimization was performed using the steepest descent algorithm, reducing potential energy fluctuations to less than 1000 kJ/mol/nm. Thermodynamic stability was ensured by equilibration under both NVT and NPT conditions at 310 K. Subsequent production simulations were performed over 100 nanoseconds using GROMACS 2023.1. These simulations focused on evaluating the stability, interactions, and therapeutic potential of *S. enterica* serovar Typhi-Gyrase B with 5-O-caffeoylquinic acid, *S. enterica* serovar Typhi - Gyrase A with isoorientin, *S. aureus*-MurE with isoorientin, *L. monocytogenes*-PrfA with 5-O-caffeoylquinic acid, *L. monocytogenes*-PrfA with isoorientin, *C. albicans*-XOG1 with ellagic acid, *C. albicans*-XOG1 with isoorientin, *B. cereus*-DltA with 5-O-caffeoylquinic acid, and *B. cereus*-DltA with isoorientin complexes. To further quantify ligand-binding affinity and complex stability, MM/PBSA free energy calculations were performed using the gmx_MMPBSA tool (Miller III et al., 2012; Valdés-Tresanco et al., 2021). The integration of

these MD simulations with MM/PBSA calculations provides a comprehensive understanding of ligand-protein interactions, facilitating the identification of potential inhibitors that exhibit both strong binding affinity and stability.

References

1. Adasme MF, Linnemann KL, Bolz SN, Kaiser F, Salentin S et al. (2021) PLIP 2021: Expanding the scope of the protein–ligand interaction profiler to DNA and RNA. Nucleic Acids Res 49(W1):W530–W534. doi: 10.1093/nar/gkab294
2. Alexander JAN, Chatterjee SS, Hamilton SM, Eltis LD, Chambers HF et al. (2018) Structural and kinetic analyses of penicillin-binding protein 4 (PBP4)-mediated antibiotic resistance in *Staphylococcus aureus*. J Biol Chem 293(51):19854–19865. doi: 10.1074/jbc.ra118.004952
3. Barna TM, Khan H, Bruce NC, Barsukov I, Scrutton NS et al. (2001) Crystal structure of pentaerythritol tetranitrate reductase: Flipped binding geometries for steroid substrates in different redox states of the enzyme. J Mol Biol 310(2):433–447. doi: 10.1006/jmbi.2001.4779
4. Belousoff MJ, Eyal Z, Radjainia M, Ahmed T, Bamert RS et al. (2017) Structural basis for linezolid binding site rearrangement in the *Staphylococcus aureus* ribosome. mBio 8(3):e00395-17. doi: 10.1128/mBio.00395-17
5. Borovinskaya MA, Pai RD, Zhang W, Schuwirth BS, Holton JM et al. (2007) Structural basis for aminoglycoside inhibition of bacterial ribosome recycling. Nat Struct Mol Biol 14(8):727–732. doi: 10.1038/nsmb1271
6. Bublitz M, Holland C, Sabet C, Reichelt J, Cossart P et al. (2008) Crystal structure and standardized geometric analysis of InlJ, a listerial virulence factor and leucine-rich repeat protein with a novel cysteine ladder. J Mol Biol 378(1):87–96. doi: 10.1016/j.jmb.2008.01.100

7. Carfi A, Duée E, Galleni M, Frère JM, Dideberg O (1998) 1.85 Å resolution structure of the zinc (II) beta-lactamase from *Bacillus cereus*. Acta Cryst *D* 54:313–323. doi: 10.1107/S0907444997010627
8. Caveney NA, Caballero G, Voedts H, Niciforovic A, Worrall LJ et al. (2019) Structural insight into YcbB-mediated beta-lactam resistance in *Escherichia coli*. Nat Commun 10:1849. doi: 10.1038/s41467-019-09507-0
9. Cleasby A, Wonacott A, Skarzynski T, Hubbard RE, Davies GJ et al. (1996) The X-ray crystal structure of phosphomannose isomerase from *Candida albicans* at 1.7 Å resolution. Nat Struct Biol 3(5):470–479. doi: 10.1038/nsb0596-470
10. Dennis ML, Lee MD, Harjani JR, Ahmed M, DeBono AJ et al. (2018) 8-Mercaptoguanine derivatives as inhibitors of dihydropteroate synthase. Chem Eur J 24(8):1922–1930. doi: 10.1002/chem.201704730
11. Ebrecht AC, van der Bergh N, Harrison STL, Smit MS, Sewell BT et al. (2019) Biochemical and structural insights into the cytochrome P450 reductase from *Candida tropicalis*. Sci Rep 9:20088. doi: 10.1038/s41598-019-56516-6
12. Fibriansah G, Kovács ÁT, Pool TJ, Boonstra M, Kuipers OP et al. (2012) Crystal structures of two transcriptional regulators from *Bacillus cereus* define the conserved structural features of a PadR subfamily. PLOS ONE 7(11):e48015. doi: 10.1371/journal.pone.0048015
13. Franceschini S, Fedkenheuer M, Vogelaar NJ, Robinson HH, Sobrado P et al. (2012) Structural insight into the mechanism of oxygen activation and substrate selectivity of flavin-dependent N-hydroxylating monooxygenases. Biochemistry 51(36):7043–7045. doi: 10.1021/bi301072w
14. Gordon E, Flouret B, Chantalat L, van Heijenoort J, Mengin-Lecreulx D et al. (2001) Crystal structure of UDP-N-acetylmuramoyl-L-alanyl-D-glutamate: meso-

diaminopimelate ligase from *Escherichia coli*. J Biol Chem 276(14):10999–11006. doi: 10.1074/jbc.M009835200

15. Gupta D, Tiwari P, Haque MA, Sachdeva E, Hassan MI et al. (2021) Structural insights into the transient closed conformation and pH-dependent ATPase activity of *Salmonella Typhi* GyraseB N-terminal domain. Arch Biochem Biophys 701:108786. doi: 10.1016/j.abb.2021.108786
16. Hampele IC, D'Arcy A, Dale GE, Kostrewa D, Nielsen J et al. (1997) Structure and function of the dihydropteroate synthase from *Staphylococcus aureus*. J Mol Biol 268(1):21–30. doi: 10.1006/jmbi.1997.0944
17. Hargrove TY, Friggeri L, Wawrzak Z, Qi A, Hoekstra WJ et al. (2017) Structural analyses of *Candida albicans* sterol 14α-demethylase complexed with azole drugs address the molecular basis of azole-mediated inhibition of fungal sterol biosynthesis. J Biol Chem 292(16):6728–6743. doi: 10.1074/jbc.M117.778308
18. Hayashi I, Oda T, Sato M, Fuchigami S (2018) Cooperative DNA binding of the plasmid partitioning protein TubR from the *Bacillus cereus* pXO1 plasmid. J Mol Biol 430(24):5015–5028. doi: 10.1016/j.jmb.2018.11.001
19. Jo S, Kim T, Iyer VG, Im W (2008) CHARMM-GUI: A web-based graphical user interface for CHARMM. J Comput Chem 29(11):1859–1865. doi: 10.1002/jcc.20945
20. Juvvadi PR, Fox D, Bobay BG, Hoy MJ, Gobeil SMC et al. (2019) Harnessing calcineurin–FK506–FKBP12 crystal structures from invasive fungal pathogens to develop antifungal agents. Nat Commun 10:4275. doi: 10.1038/s41467-019-12199-1
21. Kim HT, Kim S, Na BK, Chung J, Hwang E et al. (2017) Structural insights into the dimer–tetramer transition of FabI from *Bacillus anthracis*. Biochem Biophys Res Commun 493 (1): 28–33. doi: 10.1016/j.bbrc.2017.09.084

22. Köster S, van Pee K, Hudel M, Leustik M, Rhinow D et al. (2014) Crystal structure of listeriolysin O reveals molecular details of oligomerization and pore formation. Nat Commun 5:3690. doi: 10.1038/ncomms4690

23. Kulén M, Lindgren M, Hansen S, Cairns AG, Grundström C et al. (2018) Structure-based design of inhibitors targeting PrfA, the master virulence regulator of *Listeria monocytogenes*. J Med Chem 61(9):4165–4175. doi: 10.1021/acs.jmedchem.8b00289

24. Lafitte D, Lamour V, Tsvetkov PO, Makarov AA, Klich M et al. (2002) DNA gyrase interaction with coumarin-based inhibitors: The role of the hydroxybenzoate isopentenyl moiety and the 5′-methyl group of the noviose. Biochemistry 41(23):7217–7223. doi: 10.1021/bi0159837

25. Lockhart DE, Schuettelkopf A, Blair DE, van Aalten DMF (2014) Screening-based discovery of *Aspergillus fumigatus* plant-type chitinase inhibitors. FEBS Lett 588(17):3282–3290. doi: 10.1016/j.febslet.2014.07.015

26. Lu J, Patel S, Sharma N, Soisson SM, Kishii R et al. (2014) Structures of kibdelomycin bound to *Staphylococcus aureus* GyrB and ParE showed a novel U-shaped binding mode. ACS Chem Biol 9(9):2023–2031. doi: 10.1021/cb5001197

27. Maier JA, Martinez C, Kasavajhala K, Wickstrom L, Hauser KE et al. (2015) ff14SB: Improving the accuracy of protein side chain and backbone parameters from ff99SB. J Chem Theory Comput 11(8):3696–3713. doi: 10.1021/acs.jctc.5b00255

28. Miller BR, McGee TD, Swails JM, Homeyer N, Gohlke H et al. (2012) MMPBSA.py: An efficient program for end-state free energy calculations. J Chem Theory Comput 8(9):3314–3321. doi: 10.1021/ct300418h

29. Moon JH, Park JK, Kim EE (2005) Structure analysis of peptide deformylase from *Bacillus cereus*. Proteins 61(1):217–220. doi: 10.1002/prot.20526

30. Morris GM, Huey R, Lindstrom W, Sanner MF, Belew RK et al. (2009) AutoDock4 and AutoDockTools4: Automated docking with selective receptor flexibility. J Comput Chem 30(16):2785–2791. doi: 10.1002/jcc.21256

31. Moser J, Gerstel B, Meyer JE, Chakraborty T, Wehland J et al. (1997) Crystal structure of the phosphatidylinositol-specific phospholipase C from the human pathogen *Listeria monocytogenes*. J Mol Biol 273(1):269–282. doi: 10.1006/jmbi.1997.1290

32. Ooi A, Hussain S, Seyedarabi A, Pickersgill RW (2006) Structure of internalin C from *Listeria monocytogenes*. Acta Crystallogr *D* Struct Biol 62:1287–1293. doi: 10.1107/S0907444906026746

33. Osman KT, Du L, He Y, Luo Y (2009) Crystal structure of *Bacillus cereus* D-alanyl carrier protein ligase (DltA) in complex with ATP. J Mol Biol 388(2):345–355. doi: 10.1016/j.jmb.2009.03.040

34. Redrado-Hernández S, Macías-León J, Castro-López J, Sanz AB, Dolader E et al. (2024) Broad protection against invasive fungal disease from a nanobody targeting the active site of fungal β-1,3-glucanosyltransferases. Angew Chem Int Ed 63(34):e202405823. doi: 10.1002/anie.202405823

35. Sachdeva E, Kaur G, Tiwari P, Gupta D, Singh TP et al. (2020) The pivot point arginines identified in the β-pinwheel structure of C-terminal domain from *Salmonella typhi* DNA gyrase A subunit. Sci Rep 10:7817. doi: 10.1038/s41598-020-64792-w

36. Sawaya MR, Kraut J (1997) Loop and subdomain movements in the mechanism of *Escherichia coli* dihydrofolate reductase: Crystallographic evidence. Biochemistry 36(3):586–603. doi: 10.1021/bi962337c

37. Schubert WD, Urbanke C, Ziehm T, Beier V, Machner MP et al. (2002) Structure of internalin, a major invasion protein of *Listeria monocytogenes*, in complex with its

human receptor E-cadherin. Cell 111(6):825–836. doi: 10.1016/S0092-8674(02)01136-4

38. Sogabe S, Masubuchi M, Sakata K, Fukami TA, Morikami K, Shiratori Y, Ebiike H, Kawasaki K, Aoki Y, Shimma N, D'Arcy A, Winkler FK, Banner D, Ohtsuka T (2002) Crystal structures of *Candida albicans* N-myristoyltransferase with two distinct inhibitors. Chem Biol 9(10):1119–1128. doi: 10.1016/S1074-5521(02)00240-5
39. Sundaram R, Manohar K, Patel SK, Acharya N, Vasudevan D (2021) Structural analyses of PCNA from the fungal pathogen *Candida albicans* identify three regions with species-specific conformations. FEBS Lett 595(9):1328–1349. doi: 10.1002/1873-3468.14055
40. Sychantha D, Little DJ, Chapman RN, Boons GJ, Robinson H et al. (2018) PatB1 is an O-acetyltransferase that decorates secondary cell wall polysaccharides. Nat Chem Biol 14(1):79–85. doi: 10.1038/nchembio.2509
41. Symersky J, Monod M, Foundling SI (1997) High-resolution structure of the extracellular aspartic proteinase from *Candida tropicalis* yeast. Biochemistry 36(42):12700–12710. doi: 10.1021/bi970613x
42. Trott O, Olson AJ (2010) AutoDock Vina: Improving the speed and accuracy of docking with a new scoring function, efficient optimization and multithreading. J Comput Chem 31(2):455–461. doi: 10.1002/jcc.21334
43. Valdés-Tresanco MS, Valdés-Tresanco ME, Valiente PA, Moreno E (2021. gmx_MMPBSA: A new tool to perform end-state free energy calculations with GROMACS. J Chem Theory Comput 17(10):6281–6291. doi: 10.1021/acs.jctc.1c00645

44. Wang L, Shao X, Zhong T, Wu Y, Xu A et al. (2021) Discovery of a first-in-class CDK2 selective degrader for AML differentiation therapy. Nat Chem Biol 17(5):567–575. doi: 10.1038/s41589-021-00742-5

45. Yamasaki S, Nakashima R, Sakurai K, Baucheron S, Giraud E et al. (2019) Crystal structure of the multidrug resistance regulator RamR complexed with bile acids. Sci Rep 9:177. doi: 10.1038/s41598-018-36025-8

46. Zhang Y, Wei W, Fan J, Jin C, Lu L et al. (2020) *Aspergillus fumigatus* mitochondrial acetyl coenzyme A acetyltransferase as an antifungal target. Appl Environ Microbiol 86 (7): e02986-19. doi: 10.1128/AEM.02986-19

Table S1. Retention time, molecular ion and major MS^2 fragments with collision energies of all tested compounds.

Compounds	Retention time (min)	Molecular ion (*m/z*)	Fragment ions (m/z), (collision energy (*eV*))
3-*O*-Caffeoylquinic acid	5.00	353.101	191.30 (30)
5-*O*-Caffeoylquinic acid	5.67	353.103	191.28 (25)
Caffeic acid	6.18	179.004	134.00 (13); 135.00 (16)
Isoorientin	6.42	447.018	327.05 (27); 357.06 (23)
Rutin	6.71	609.197	299.98 (42); 301.20 (32)
Vitexin	6.81	430.999	311.06 (27); 283.06 (36)
Quercetin 3-*O*-glucoside	6.96	463.100	271.00 (43); 300.00 (30)
p-Coumaric acid	6.97	163.031	93.12 (39): 119.09 (16)
Ellagic acid	7.07	300.980	284.06 (33): 300.04 (30)
Isorhamnetin-3-O-rutinoside	7.13	622.827	313.95 (7): 271.20 (21)
Kaempferol 3-*O*-glucoside	7.40	447.008	255.03 (43); 284.03 (29)
Eriodictyol	8.62	286.974	150.93 (19); 135.02 (32)
Luteolin	8.70	285.035	133.05 (30); 150.95 (24)
Naringenin	9.39	271.036	119.10 (25); 151.07 (19)
Hispidulin	9.57	299.060	284.00 (21); 256.00 (25)

Table S2. The docking score (kcal/mol) and interacting residues of the enzyme and protein.

Compound	Target	PDB ID	Binding energy (kcal/mol)	RMSD	Interaction		Binding site
					Type	Number	
Ellagic acid	*S. enterica* serovar Typhi-Gyrase B	5ZXM	-9.6	1.0647	Hbond	4	ASN A:46;ASP A:73;ARG A:76;GLY A:77
5-O-Caffeoylquinic acid	*S. enterica* serovar Typhi-Gyrase B	5ZXM	-9.1	1.0209	Hbond	10	ASN A:46;GLU A:50;ASP A:73;GLY A:77;GLY A:102;LYS A:103;TYR A:109;LEU A:115;GLY A:117;THR A:165
Isoorientin	*S. enterica*-Gyrase A	5ZTJ	-9.8	0.8215	Hbond	14	HIS A:545;ASP A:579;ARG A:580;ARG A:580;ARG A:630;THR A:632;ASP A:686;ASP A:686;SER A:734;ILE A:737;ARG A:739;ARG A:739;GLN A:788;ARG A:838
Isoorientin	*S. aureus*-PBP2	5TW8	-9.2	0.2521	Hbond	9	SER A:75;LYS A:78;GLU A:114;ASN A:141;THR A:180;THR A:180;ALA A:182;GLU A:183;SER A:262
Ellagic acid	*S. aureus*-MurE	4C13	-9.1	nd	Hbond	9	THR A:111;GLY A:113;LYS A:114;LYS A:114;THR A:115;THR A:115;SER A:116;HIS A:353;THR A:354
5-O-Caffeoylquinic acid	*S. aureus*-MurE	4C13	-9.4	nd	Hbond	8	THR A:111;LYS A:114;THR A:115;THR A:115;SER A:116;ASP A:204;HIS A:205;HIS A:353
Isoorientin	*S. aureus*-MurE	4C13	-10.4	0.2157	Hbond	13	LYS A:114;THR A:115;SER A:116;GLU A:177;SER A:180;HIS A:181;HIS A:181;ASP A:204;HIS A:205;ARG A:335;ARG A:335;ASP A:350;HIS A:353
5-O-Caffeoylquinic acid	*L. monocytogenes*-PrfA	6EXL	-9	0.5254	Hbond	26	PHE A:225;PHE A:225;THR A:232;THR A:232;TRP A:233;TRP A:233;;;TYR A:62;TYR A:62;TYR A:126;TYR A:126;TYR A:126;TYR A:126;ILE A:149;ILE A:149;TYR A:154;TYR A:154;;;LYS A:64;LYS A:64;GLN A:146;GLN A:146;LEU A:150;LEU A:150
Isoorientin	*L. monocytogenes*-PrfA	6EXL	-9.9	0.7025	Hbond	28	PHE A:225;PHE A:225;THR A:232;THR A:232;TRP A:233;TRP A:233;ASN A:2;ASN A:2;ASN A:2;ASN A:2;GLN A:61;GLN A:61;TYR A:62;TYR A:62;TYR A:62;TYR A:62;LYS A:64;LYS A:64;LYS A:64;LYS A:64;LYS A:64;LYS A:64;GLN A:118;GLN A:118;TYR A:126;TYR A:126
Isoorientin	*L. monocytogenes*-MurA	3R38	-9	0.9897	Hbond	11	ALA A:94;ARG A:122;ARG A:122;SER A:163;SER A:163;VAL A:164;GLY A:165;ARG A:233;ARG A:233;MET A:297;THR A:304
5-O-Caffeoylquinic acid	*L. monocytogenes*-InlA-Pose-1	1O6T	-9	0.1256	Hbond	12	LYS A:425;LYS A:425;LYS A:425;SER A:429;SER A:429;SER A:429;SER A:429;THR A:454;THR A:454;ASP A:457;ASP A:457;ASP A:457
5-O-Caffeoylquinic acid	*L. monocytogenes*-InlA-Pose-2	1O6T	-9.1	0.8865	Hbond	11	LYS A:56;THR A:80;THR A:80;THR A:80;GLN A:82;GLN A:82;GLN A:82;ASP A:84;ASP A:84;ASP A:84;ASP A:84
Isoorientin	*E. coli*-MurE	1E8C	-9	1.0334	Hbond	8	GLY B:118;LYS B:119;THR B:120;ASN B:156;GLU B:182;SER B:184;ARG B:192;ARG B:192
Isoorientin	*C. tropicalis*-SAP	1J71	-9.1	9.3811	Hbond	7	SER A:13;THR A:88;ASP A:120;ASP A:120;GLU A:194;ARG A:196;GLY A:220
Ellagic acid	*C. tropicalis*-CPR	6T1U	-9.9	28.974	Hbond	3	TYR A:118;SER A:441;ASP A:677
5-O-Caffeoylquinic acid	*C. tropicalis*-CPR	6T1U	-9	22.661	Hbond	7	SER A:78;VAL A:91;THR A:340;THR A:340;ILE A:429;GLU A:430;HIS A:434
Isoorientin	*C. tropicalis*-CPR	6T1U	-9.7	20.908	Hbond	7	THR A:67;THR A:67;TYR A:118;GLY A:119;GLY A:121;ASN A:160;TYR A:439
Ellagic acid	*C. albicans*-XOG1	2PBO	-9.7	0.4147	Hbond	5	ASP A:145;ASP A:145;ASN A:146;GLU A:192;ARG A:309
5-O-Caffeoylquinic acid	*C. albicans*-XOG1	2PBO	-9.3	1.0656	Hbond	4	TYR A:29;ASN A:146;TYR A:255;LEU A:304

Isoorientin	*C. albicans*-XOG1	2PBO	-10.5	0.4553	Hbond	4	ASP A:145;ASP A:145;ASP A:145;ARG A:309
Isoorientin	*C. albicans*-NMT	1IYK	-9.4	0.9627	Hbond	7	TYR A:107;TYR A:107;LEU A:177;THR A:211;THR A:211;HIS A:227;ASN A:392
5-O-Caffeoylquinic acid	*B. cereus*-DltA	3FCE	-9.1	0.672	Hbond	6	ASN A:292;THR A:293;THR A:297;TYR A:379;THR A:381;ASP A:383
Isoorientin	*B. cereus*-DltA	3FCE	-9.8	1.5018	Hbond	11	THR A:152;THR A:152;GLY A:270;LEU A:273;ASN A:292;GLY A:295;THR A:297;THR A:297;GLU A:298;TYR A:379;THR A:381
